# New Perspectives in the Noble Gas Chemistry Opened by Electrophilic Anions

**DOI:** 10.3389/fchem.2020.580295

**Published:** 2020-11-13

**Authors:** Markus Rohdenburg, Vladimir A. Azov, Jonas Warneke

**Affiliations:** ^1^Fachbereich 2-Biologie/Chemie, Institut für Angewandte und Physikalische Chemie, Universität Bremen, Bremen, Germany; ^2^Department of Chemistry, University of the Free State, Bloemfontein, South Africa; ^3^Wilhelm-Ostwald-Institut für Physikalische und Theoretische Chemie, Universität Leipzig, Leipzig, Germany; ^4^Leibniz Institute of Surface Engineering (IOM), Leipzig, Germany

**Keywords:** noble gas compounds, *closo*-dodecaborates, anionic electrophiles, mass spectrometry, collision induced dissociation, weakly coordinating cations, soft landing, DFT calculations

## Abstract

Binding of noble gases (NGs) is commonly considered to be the realm of highly reactive electophiles with cationic or at least non-charged character. Herein, we summarize our latest results evidencing that the incorporation of a strongly electrophilic site within a rigid cage-like anionic structure offers several advantages that facilitate the binding of noble gases and stabilize the formed NG adducts. The anionic superelectrophiles investigated by us are based on the *closo*-dodecaborate dianion scaffold. The record holder [B_12_(CN)_11_]^−^ binds spontaneously almost all members of the NG family, including the very inert argon at room temperature and neon at 50 K in the gas phase of mass spectrometers. In this perspective, we summarize the argumentation for the advantages of anionic electrophiles in binding of noble gases and explain them in detail using several examples. Then we discuss the next steps necessary to obtain a comprehensive understanding of the binding properties of electrophilic anions with NGs. Finally, we discuss the perspective to prepare bulk ionic materials containing NG derivatives of the anionic superelectophiles. In particular, we explore the role of counterions using computational methods and discuss the methodology, which may be used for the actual preparation of such salts.

## Introduction

The spontaneous binding of a noble gas (NG) at room temperature remains the privilege of the strongest electrophiles (Brock et al., 2013; Pan et al., 2019). Due to the closed electron shell of NGs, their electron affinities are negative and a NG atom cannot be chemically attacked by any type of nucleophile. Therefore, the only possibility to form a bond is to abstract electron density from the NG. A number of NG adducts with electrophiles have been characterized in cryogenic matrices (Khriachtchev et al., 2000; Wang and Wang, 2013; Wang X. et al., 2013) where even very weak bonds can hold these compounds together. Under these reaction conditions, it was even possible to observe the first neutral argon compound (Khriachtchev et al., 2000; Bochenkova et al., 2009). In contrast, room temperature NG chemistry is mainly limited to the heavier NGs, mainly xenon (Malm et al., 1965; Haner and Schrobilgen, 2015) and, on a much more limited basis, krypton (Lehmann et al., 2002). The majority of NG compounds contains fluorine or oxygen (Liebman and Deakyne, 2003; Samanta, 2014) since these elements have very high electronegativities and are strongly electron withdrawing. Also, compounds with vacant boron and beryllium atoms were often proposed as suitable NG binders due to their exceptional electron deficiency (Pan et al., 2014; Saha et al., 2016, 2019). It is also well understandable that strong NG bonds have been frequently reported for highly reactive isolated cations (Grandinetti, 2011), which satisfy their demand for electrons with almost everything that comes into reach—even a NG. However, other molecules or atoms, which are more nucleophilic, tend to substitute the NG and bind the electrophilic cations. Using cationic NG derivatives for the generation of a condensed phase compound is therefore hindered by a fundamental problem: a cation needs to be paired with a counteranion. Anions are typically much stronger nucleophiles than the NG itself and, therefore, immediately substitute the NG by forming a bond with the electrophilic binding site. Experimental access to most reported molecular NG cations, which are stable at room temperature, remains limited to the low-pressure gas phase of a mass spectrometer.

Recently, we discovered that the electrophilic anion [B_12_Cl_11_]^−^ is able to bind spontaneously xenon and krypton at room temperature in the gas phase (Rohdenburg et al., 2017). Later, aiming at further increase of reactivity, we prepared and investigated the cyanated derivative [B_12_(CN)_11_]^−^, which was found to be much more electrophilic than its predecessor. It is able to bind argon at room temperature (Mayer et al., 2019) and can even form a stable adduct with extremely unreactive neon (Mayer et al., 2020) at temperatures up to 50 K. High level computational studies and investigations of the weak complexes in the cold matrix have shown that the binding energies for Ne are often even smaller than for He in similar adducts and, therefore, Ne was discussed to be the most inert NG (Frenking et al., 1990; Grandinetti, 2013; Grochala, 2018). These electrophilic anions are generated by collision induced dissociation (CID) of their gaseous precursors *closo*-dodecaborate dianions [B_12_X_12_]^2−^ (X=halogen, CN), which constitute a class of very stable and inert weakly coordinating anions (WCAs) (Knapp, 2013). The discussed experimental results inspired further theoretical investigations on the NG binding properties of similar compounds, even with higher negative charge (Joshi and Ghanty, 2019, 2020).

In the first section of this perspective, we summarize and explain the binding concepts of electrophilic anions with NGs and their advantages when compared with “common” cationic electrophiles. Then, we discuss open questions that still need to be clarified to obtain a comprehensive and quantitative understanding of the binding between electrophilic anions and NGs. Finally, we deepen the insights on the effect of counterions on the NG bond with electrophilic anions and discuss the possibilities and limitations for the preparation of salts using [B_12_X_11_NG]^−^ anions as precursors.

## Results and Discussion

**Remark**: If not stated otherwise, calculations were performed using the B3LYP DFT functional and, in most cases, results have not been confirmed using high-level correlated methods. Reported numbers are given for the purpose of qualitative comparison of reactions and should not be interpreted as quantitative thermochemistry values. More details can be found in the methods section and in the Supplementary Material.

### Fundamental Concepts of Electrophilic Anions and Their Reactivity

Several fundamental concepts underline the formation and binding traits of electrophilic anions. We will discuss them below on the example of *closo*-dodecaborate dianions [B_12_X_12_]^2−^, which have been the objects of our detailed investigations. However, related molecular systems, for example so called anionic “super atomic clusters” (Jena and Sun, 2018) or other *closo*-borate anions may show similar features (Rohdenburg et al., 2020).

#### (i) Breaking the Most Stable Generates the Most Reactive

**Figure 1:**
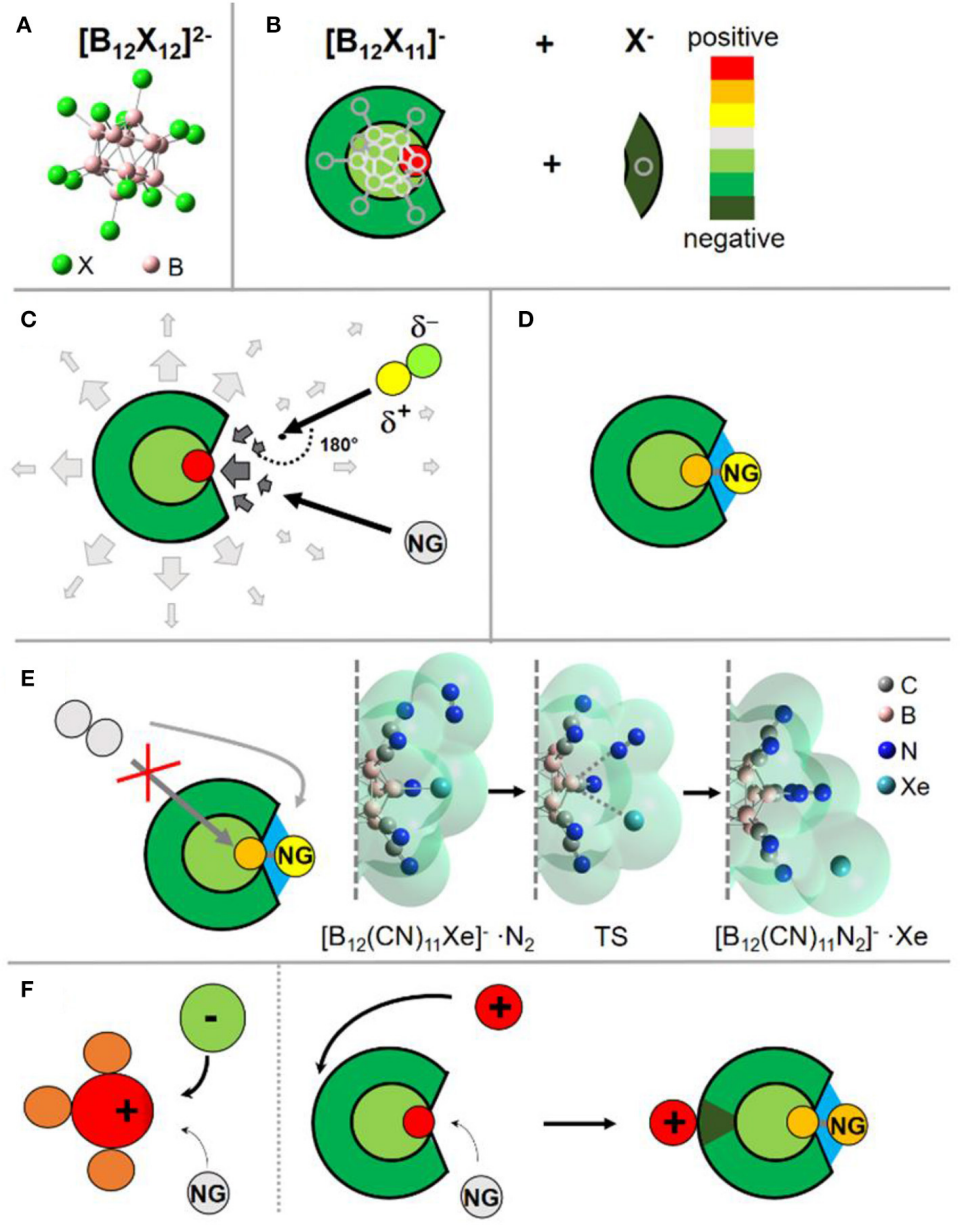
Visualization of the discussed binding concept. **(A)** Structure of the *closo*-dodecaborate dianions. **(B)** Simplified scheme to show the charge distribution in the fragment [B_12_X_11_]^−^ after abstraction of a negatively charged substituent X^−^ from the doubly charged precursor. **(C)** Schematic visualization of the electric field near the ion. Close to the binding site the field changes its direction, leading to the change of the preferred orientation for a polar reaction partner. **(D)** Schematic visualization of the ion-NG interaction. Next to the dative bond to the boron (gray), interactions with the surrounding substituents (electrostatics, dispersion) are shown in blue. **(E)** S_N_2 substitution by another nucleophile is blocked from the back; a nucleophile must attack from the side, as shown on an example of the substitution reaction of Xe with N_2_ in [B_12_(CN)_11_Xe]^−^ adduct. The molecular surface is plotted semitransparent to show the steric demand of the reaction partners. The start and end structures are true minima. For detailed discussion, see Concept (vi). **(F)** Schematic explaining the different effect of counterions on NG binding in an electrophilic cation and an electrophilic anion. While the case of an electrophilic cation NG and counterion compete for one binding site, they target different binding sites in the case of the electrophilic anion. The countercation increases the strength of the formed B-NG bond, see discussion in Concept (vii). Images are partially based on our previous illustrations (Rohdenburg et al., 2017; Mayer et al., 2019, 2020).

An exceptionally reactive electrophilic site within an ion is required to bind a NG atom. In our search for very reactive molecular ions we were following a simple idea: breaking bonds in very stable molecular ions should result in the most reactive fragments. A large driving force should be present in the fragment to recover the original highly stable structural and electronic configuration of the precursor. The *closo*-dodecaborate dianions [B_12_X_12_]^2−^ (Figure 1A) possess very high icosahedral (I_h_) molecular symmetry, a 3D-aromatic σ-electron system and exceptional electronic stability (Warneke et al., 2017). As first predicted theoretically (Zhao et al., 2016) and later confirmed experimentally (Mayer et al., 2019), the [B_12_(CN)_12_]^2−^ dianion has the highest second electron binding energy (the energy necessary to detach an electron from the doubly charged anion) known for small multiply charged anions synthesized so far (>5 eV). The [B_12_Cl_12_]^2−^ dianion has been used to stabilize extremely reactive countercations in the condensed phase, including methyl (Bolli et al., 2010) and silyl (Kessler et al., 2010) cations, thus evidencing its exceptional chemical inertness. These facts demonstrate the outstanding electronic, structural, and chemical stability of *closo*-dodecaborate dianions and make them ideal precursors for the generation of highly reactive fragments by breaking a B-X bond.

#### (ii) Positive, Strong Electrophilic Site Within an Anion

Since only strong electrophiles, but not nucleophiles, are able to bind a NG by forming a bond with significant covalent character, anions have raised limited attention as potential strong NG binders. However, the fragment [B_12_X_11_]^−^ possess a special charge distribution: although the ion is overall negatively charged, the vacant boron atom exhibits a strong positive partial charge (Figure 1B), very electrophilic in nature and, therefore, can bind a NG atom. Experimentally, the positive site was probed by infrared photodissociation spectroscopy [for details on the used instrument and method, see Heine and Asmis (2015)] of its CO adduct (Gruene et al., 2008). The CO stretching frequency is a sensitive probe for the charge state of a binding site. Thus, a CO molecule was allowed to bind to the anion and IR spectra of [B_12_X_11_CO]^−^ were measured in the gas phase. A strong blue shift of the CO stretching frequency, typically observed for very electrophilic cations (Saha et al., 2017) like, for example, the phenyl cation (Winkler and Sander, 2006), was measured for the [B_12_X_11_CO]^−^ ions. Computationally, the picture of a positive vacant boron atom was confirmed by calculated atomic charges, the electrostatic potential and the electric field. The calculated charge distributions of [B_12_X_11_]^−^ suggests that, from a qualitative point of view, this anion could better be described as a reactive cation embedded into an inert dianionic framework. The leaving X^−^, which is disconnected from the [B_12_X_12_]^2−^ precursor, takes the electron density almost exclusively from the boron atom to which it was bound and very limited charge redistribution occurs in the formed fragment ion. The analysis of the electron localizability indicator (ELI-D) evidenced that the vacant boron site of [B_12_X_11_]^−^ is electronically separated from the delocalized σ-electron system, which gives an explanation to the strong electrophilic and cationic nature of this vacant boron site (Figure 1B) (Rohdenburg et al., 2017).

#### (iii) Preserving the Reactive Site Within a Large Molecular Framework

Large ion sizes are an advantage for the observation of weakly bound adducts in the gas phase. Upon collision of the ion with a NG, a “hot collision complex” is formed. If the collisional energy can be efficiently redistributed into many vibrational degrees of freedom, the complex can survive until further collisions with background gases cool the adduct. Collision complexes of smaller ions have much less chances to survive long enough for experimental observation, even if the NG bond may have a similar strength. However, most highly reactive isolated cations which exhibit NG binding at room temperature are small (<10 atoms). Often, a highly reactive positively charged site embedded into a large molecular framework results in structural rearrangement of the molecular ion. For example, electrophilicity of the phenyl cation can be increased by the substitution of all five hydrogens by fluorine, raising its binding energy with Xe theoretically from around 70 to 150 kJ/mol. However, it has been experimentally and theoretically shown that, unlike the phenyl cation [C_6_H_5_]^+^, the pentafluorinated phenyl cation [C_6_F_5_]^+^ is unstable in the gas phase and spontaneously fragments by expulsion of difluorocarbene. This rearrangement results in a more stable cationic species containing an aromatic cyclopropenium moiety (Wang H.-Y. et al., 2013), which has only a small Xe binding enthalpy of around 15 kJ/mol. Though pentafluorinated phenyl cations cannot be generated in a gas phase reaction, the preparation of its xenon derivative, the [C_6_F_5_Xe]^+^ cation, was possible in a reaction between XeF_2_ and B(C_6_F_5_), proceeding via a concerted substitution mechanism, at low temperature in an inert organic solvent, such as CH_2_Cl_2_ or MeCN (Frohn and Jakobs, 1989; Naumann and Tyrra, 1989). In our case, the rigid B_12_ framework does not allow for any intramolecular rearrangements of the [B_12_X_11_]^−^ anion, independent on the nature of the substituent X, and can be used as a non-solvated, isolated ion for binding of NG atoms. Comprising 23 atoms, [B_12_X_11_]^−^ is large in comparison to most experimentally observed NG binding ions, thus affording efficient stabilization of collisional complexes.

#### (iv) Preference for Binding of Non-polar Nucleophiles

A cationic binding site within an anion results in a special distribution of the electric field near this site. Reaction partners located far away from the ions are only affected by the anionic negative charge, while close to the positive binding site, the electric field changes its direction. Polar molecules such as water, which are usually stronger nucleophiles than NGs, change their preferred orientation upon approach to the binding site (Figure 1C). This situation may result in substantial centrifugal barriers. In contrast, a NG atom can just repolarize and may have an advantage in the competition with polar nucleophiles for the binding site. Thus, such electrophilic anions should show relative selectivity in binding to non-polar nucleophilic species.

#### (v) Large Interaction Surface Between Ion and NG

The binding site of an electrophilic anion [B_12_X_11_]^−^ is located within a “crater” of five substituents X. Therefore, beside the dative bond formed by the shift of electron density from the NG to the electrophilic boron, a large surface for additional electrostatic forces and dispersion interactions between the anion and a NG atom strengthens the total interaction (Figure 1D). Upon formation of the dative bond with [B_12_X_11_]^−^, the NG atom becomes partially positively charged, which affords additional attractive electrostatic interaction with the negatively charged [B_12_X_11_]^−^ residue. Dispersion interactions (Wagner and Schreiner, 2015) are in particular strong for the heavier and more polarizable NGs, but they also make a significant contribution in the case of Ar and Ne binding (Mayer et al., 2019, 2020).

#### (vi) Protection of the Adducts Against Substitution

The cage structure of the borate anion protects the B-Ng bond against a typical back-side nucleophilic attack following the S_N_2 mechanism. Therefore, only a side attack of the B-NG bond by a nucleophile is possible. A significant elongation of the B-NG bond is required before such a nucleophile will be able to interact with the vacant boron atom. Therefore, we expect the energy of the transition state (TS) for substitution to be strongly correlated with the B-NG dissociation enthalpy (DE). As an example, we have computationally investigated the substitution of Xe in [B_12_(CN)_11_Xe]^−^ by the stronger nucleophile N_2_. Xe is bound to [B_12_(CN)_11_]^−^ with a 0 K DE of 114 kJ/mol. We started with a {[B_12_(CN)_11_Xe]^−^···N_2_} complex, in which N_2_ is only weakly bound to the ion (Figure 1E). Then, we simulated the attack by systematically reducing the B-N distance. The optimized TS was found to be 90 kJ/mol higher in enthalpy than the initial minimum with a B-Xe bond significantly elongated by 1.3 Å. The product of the favorable substitution was found at ΔH_0K_ = −52 kJ/mol. For direct comparison, we choose the adduct between Xe and ${\text{CH}}_{3}^{+}$, which binds Xe even stronger (see details in Supplementary Material, section Substitution of Xe by N_2_ in [H_3_CXe]^+^). Although the substitution of Xe in [H_3_CXe]^+^ with N_2_ is less exothermic (ΔH_0K_ = −20 kJ/mol), the TS was calculated to be considerably lower in enthalpy with ΔH_0K_ = 17 kJ/mol and a C-Xe bond length elongation by 0.5 Å was found. This demonstrates that the TS for substitution reactions in [B_12_(CN)_11_NG]^−^ is comparatively high. We note that the same argumentation qualitatively holds when repeating the calculations on the BMK-GD3BJ/aug-cc-pVTZ (SDD) level of theory (see details in Supplementary Material, section Substitution of Xe by N_2_ on BMK-GD3BJ/aug-cc-pVTZ (SDD) Level of Theory). It has been reported that the BMK functional yields better quantitative thermochemistry values for NG compounds compared to our standard approach relying on the dispersion-corrected B3LYP DFT functional (Grandinetti, 2018). The dispersion-corrected B3LYP functional showed sufficient accord with high-level post-Hartree Fock methods like SCS-MPS or CCSD(T) in our former studies (Rohdenburg et al., 2017; Mayer et al., 2019, 2020) of similar Ng compounds as discussed herein.

#### (vii) Stabilization of Adducts With Counterions

**Figure 2:**
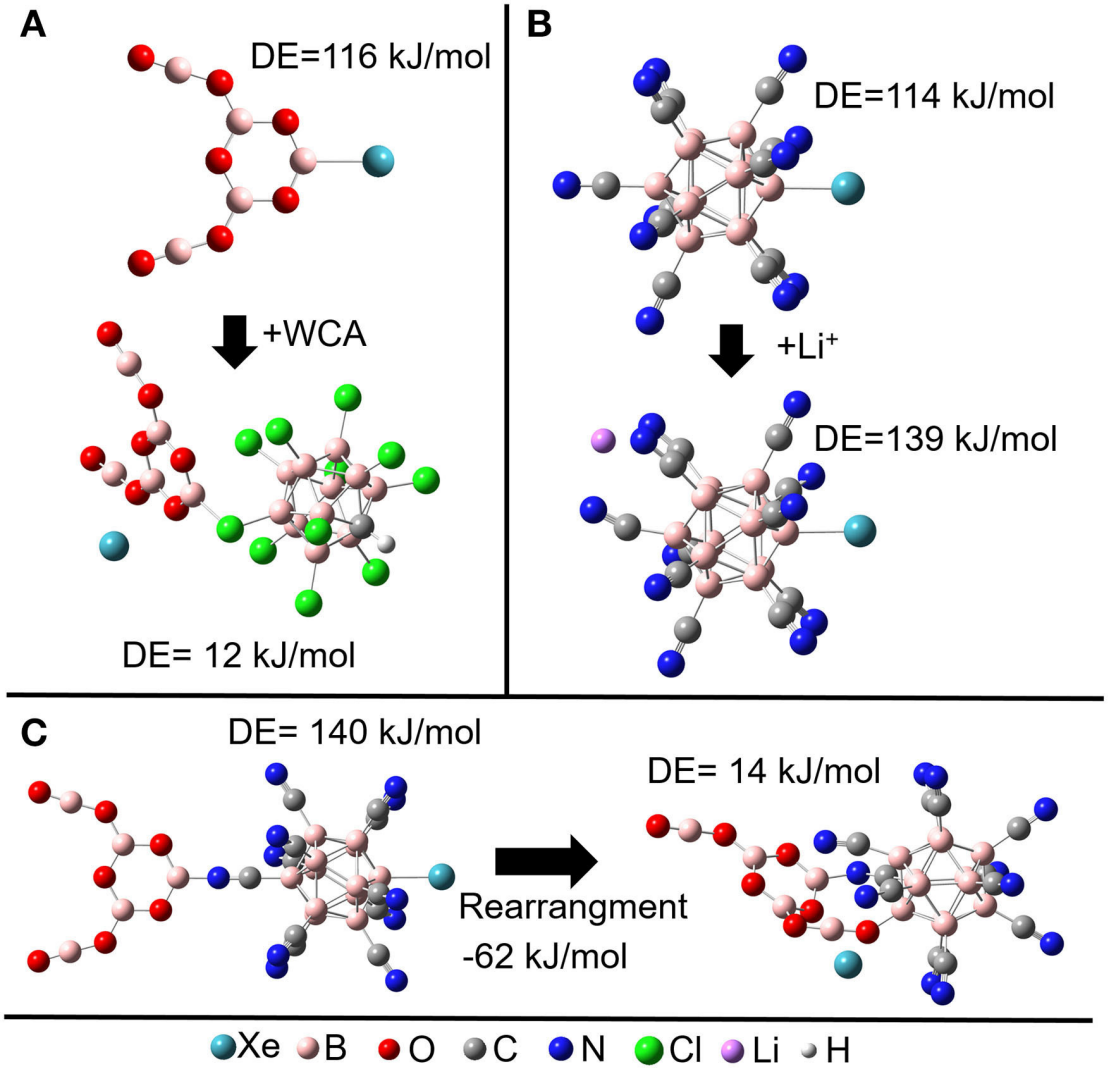
Effect of ion pairing on the 0 K dissociation enthalpy (DE) of Xe **(A)** Combinations of the WCA [HCB_11_Cl_11_]- with [B_5_O_7_Xe]^+^. **(B)** Combination of Li^+^ with [B_12_(CN)_11_Xe]^−^. **(C)** Combination of [B_5_O_7_]^+^ with [B_12_(CN)_11_Xe]^−^: a rearrangement is thermochemically preferred and results in the loss of the vacant boron site which binds the NG.

There exists an intrinsic thermodynamic problem for highly reactive cations to form stable NG adducts in the condensed phase, since they demand the presence of counterions. These counterions are anions that have commonly nucleophilic nature. Usually, anionic counterions will be by far stronger competitors for the positive electrophilic binding site of a reactive cation than NGs and a substitution of the NG by the anion's nucleophilic site will be energetically preferred. The existence of a salt consisting of a NG binding cation and a counteranion is only possible if strong kinetic barriers hinder this substitution reaction. In contrast, [B_12_X_11_NG]^−^ requires a countercation. Simple counterions like alkali cations do not show any tendency to bind to the electrophilic boron site. Instead, the most preferred position of a cation is the backside of the [B_12_X_11_NG]^−^ ion, which constitutes its most negative region (Figure 1F). In order to demonstrate this concept on an example, we compare the [B_5_O_7_Xe]^+^ cation (Jin et al., 2017), and the [B_12_(CN)_11_Xe]^−^ anion (see Figures 2A,B, respectively) using computational methods. [B_5_O_7_]^+^ belongs to the strongest singly charged cationic NG binders observed experimentally, and its binding strength toward NGs is comparable with [B_12_(CN)_11_]^−^. The 0 K DE for Xe is calculated to be around 115 kJ/mol for the [B_5_O_7_Xe]^+^ cation. If a chloride, a “simple” atomic anion, is placed close to the [B_5_O_7_Xe]^+^ cation, geometry optimization of the ion pair results in the substitution of the Xe atom from the adduct, and no other minimum can be found. Previously, the stabilization of highly labile cations has been performed using extremely weakly coordinating anions (Strauss, 1993; Krossing and Raabe, 2004; Riddlestone et al., 2018). One of the most prominent and successful examples of them is the carba-*closo*-dodecaborate anion [HCB_11_Cl_11_]^−^ (Juhasz et al., 2004). However, placing even this very inert anion next to the [B_5_O_7_Xe]^+^ cation also resulted in elimination of Xe upon geometry optimization (Figure 2A). The electrophilic boron in [B_5_O_7_]^+^ attaches to a halogen substituent and the resulting neutral unit [B_5_O_7_-HCB_11_Cl_11_] has no vacant site and, thus, a small Xe binding strength. Therefore, it appears extremely difficult to stabilize cationic NG adducts like [B_5_O_7_Xe]^+^ in the condensed phase, even using the most weakly coordinating anions known today. If instead a simple monoatomic Li^+^ cation is combined with [B_12_(CN)_11_Xe]^−^, ion pairing does not result in the elimination of the Xe from its adduct. On the contrary, the B-NG bond is additionally stabilized against dissociation by 25 kJ/mol in comparison to the isolated [B_12_(CN)_11_Xe]^−^ anion (see Figure 2B). The Coulomb attraction of the cation on the electron cloud of the anion results in further polarization and strengthens the cationic character of the vacant boron site that binds to the NG atom. The calculated NPA charge of Xe increases from +0.70 *e* in [B_12_(CN)_11_Xe]^−^ to +0.74 *e* in Li[B_12_(CN)_11_Xe]^−^, showing the enhanced electrophilic character of the anion upon ion pairing.

The stabilization of the B-NG bond upon ion pairing with a cation is based on the electron withdrawing effect of a cation. Therefore, it may be suggested that an even more electrophilic cation should have a stronger stabilizing effect on the B-NG bond. In our computational investigations, we studied the [B_5_O_7_]^+^ ion as a counterion for [B_12_(CN)_11_Xe]^−^. The exceptionally positive boron atom (NPA charge +1.5 *e*) should have an even stronger electron withdrawing effect. However, in contrast to Li^+^, [B_5_O_7_]^+^ interacts only with one CN substituent at the backside of [B_12_(CN)_11_Xe]^−^ and the stabilizing effect for the B-Xe bond is in both cases comparable (Figures 2B,C). Also, binding of [B_5_O_7_]^+^ to the backside of [B_12_(CN)_11_Xe]^−^ is not the global minimum of this ion pair. It is much more favorable to bind the vacant boron atom of the anion [B_12_CN_11_]^−^ to an oxygen of [B_5_O_7_]^+^ while the vacant boron atom of the cation binds to a nitrogen atom of a CN substituent of the anion, (Figure 2C). This releases the Xe atom and demonstrates a potential problem for the use of molecular cations with nucleophilic binding sites. This problem is avoided when atomic cations like Li^+^ and Na^+^ are used.

To sum up, the unique combination of multiple factors discussed above render halogenated/cyanated *closo*-dodecaborate fragment ions [B_12_X_11_]^−^ special properties that allow them to selectively bind NGs and other weakly nucleophilic substances and additionally stabilize these adducts kinetically. In principle, applicability of these concepts can be extended to other compounds with similar structural and electronic properties, such as diverse anionic cage borates or metal clusters. However, synthetic accessibility, high chemical stability, and exceptionally high reactivity of the vacant boron site make [B_12_X_11_]^−^ fragment ions the most promising superelectrophilic anions for our further studies.

### Next Steps Toward a Comprehensive Understanding of NG Binding by Electrophilic Anions

Several parameters influence the properties of the reactive site and therefore the bond between an electrophilic anion and a NG atom. The positive partial charge of the vacant boron atom in [B_12_X_11_]^−^ (concept ii) appears to be of critical importance. We expect a correlation between the reactivity of [B_12_X_11_]^−^ and the electronic stability of the dianionic precursor [B_12_X_12_]^2−^: the more stable the dianion, the more electrophilic its monoanionic fragment [B_12_X_11_]^−^ should become. This assumption is supported by the so far limited data on different electrophilic anions that is available. However, the energetics of NG binding is not solely affected by the vacant positive boron atom, but also by the interactions of NG with the five substituents X surrounding the binding site. Their partial charge, steric demand, and polarizability (critical for dispersion forces) are expected to have a significant influence on the NG binding. We aim for a better understanding how strongly these interactions influence the NG binding in electrophilic anions. A comparative experimental and theoretical study probing the binding of different electrophilic anions [B_12_X_11_]^−^ (X = F, Cl, Br, I, CN) toward different NGs (Ne, Ar, Kr, Xe) is currently in progress and the results are expected to be published soon.

A quantitative understanding of the kinetic parameters influencing NG binding may be even more challenging. Water is a competing and much stronger nucleophile, which is usually present in significant amounts in the background of mass spectrometers at room temperature. Highly reactive cations (e.g., the phenyl cation), which are calculated to form bonds to NGs with enthalpies similar to [B_12_X_11_]^−^, did not bind the NGs under the same experimental conditions at room temperature. Instead, only the water adduct of the phenyl cation was detected (Rohdenburg et al., 2017). The better redistribution of collisional energy (see concept iii), a reduced cross section for a reactive collision of the polar water with [B_12_X_11_]^−^ (concept iv) or a better protection of the B-Ng bond against substitution with water (concept vi) may all contribute to the relative stabilization of [B_12_X_11_NG]^−^ in comparison with [C_6_H_5_NG]^+^. An evaluation, which of these points is most important is currently difficult for us. In particular, an estimation of the centrifugal barrier for binding of polar molecules with a cationic site within an anion cannot be performed using common models for ion–molecule collision theory. Complex, much more sophisticated models, which take the special electric field and steric demand of the substituents X into account, will be required.

The observation of a strong NG bond in the gas phase always leads to the question if this bond may hold together the same compound or ion in a condensed phase. The thermodynamic stabilization of the B-NG bond in [B_12_X_11_NG]^−^ by addition of a countercation (vii) underlines one of the most important features, which distinguishes electrophilic anions from NG-binding cations: a thermodynamically stable, neutral ion pair can be formed. This is certainly very promising for the attempt of generating condensed phase material. However, additional challenges arise in this case, which have not been discussed in our previous publications. The goal of the last section of this perspective is to discuss further challenges on the molecular level, which may arise from building condensed phase materials from [B_12_X_11_NG]^−^ adducts and to suggest feasible approaches to overcome these challenges.

### On the Way to Bulk Salts of the Anionic NG Derivatives

Experimentally, we consider electrospray-coupled high ion-current deposition methods, so called ion soft landing (Franchetti et al., 1977; Laskin et al., 2018), to be the most promising tool for the bulk preparation of possible salts with [B_12_X_11_NG]^−^ anions generated in the gas phase. The precursor [B_12_X_12_]^2−^ can be transferred via electrospray ionization into the gas phase, where fragmentation by CID yields [B_12_X_11_]^−^. After reaction with NG, the [B_12_X_11_NG]^−^ can be mass selected and guided to a surface to be deposited with low kinetic energy in order to preserve the relatively weak B-NG bond from decomposition. Recently introduced sequential soft-landing of anions and cations (Su et al., 2019) may be used to combine sub-monolayers of [B_12_X_11_NG]^−^ with sub-monolayers of selected cations to form bulk layers of condensed material in a stepwise manner. This approach has already been shown to be useful for the buildup of multilayer materials on surfaces with mass selected anions and cations. To ensure the success of corresponding experiments it is imperative to determine which cations should be most effective for the stabilization of the [B_12_X_11_NG]^−^ adducts in the condensed phase. We continue to use [B_12_(CN)_11_Xe]^−^ as a concrete example in the following discussion.

**Figure 3:**
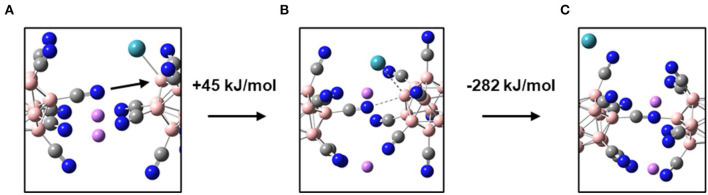
Illustration of the Xe substitution by a CN substituent of a neighboring anion within a Li[B_12_(CN)_11_Xe] dimer. **(A)** Starting geometry (true minimum), **(B)** transition state, **(C)** Product of final substitution. Extracts of the molecular structure of the dimer are shown to ensure better visibility of the reactive region. For atom assignment, please see Figure 2.

In a condensed material, the [B_12_(CN)_11_Xe]^−^ ion will not only be paired with one counterion like Li^+^, but will be closely surrounded by multiple {[B_12_(CN)_11_Xe]^−^Li^+^} units. Thus, negatively charged CN substituents of a neighboring anion will be located in spatial proximity to the electrophilic site of [B_12_X_11_NG]^−^. When considering two gaseous [B_12_(CN)_11_Xe]^−^ ions in a mass spectrometer, substitution of Xe by formation of a B-NC bond is thermochemically favored by −86 kJ/mol. In the gas phase, this reaction has never been observed because a large Coulomb barrier kinetically hinders two [B_12_X_11_NG]^−^ anions from approaching each other. In the bulk of a salt with closely spaced {[B_12_X_11_NG]^−^Li^+^} ion pairs, the long-range repulsive separation force between two [B_12_X_11_NG]^−^ anions can be almost neglected due to the presence of charge compensating Li^+^ cations. We used a simplified model consisting of two {[B_12_(CN)_11_Xe]^−^Li^+^} units to estimate the TS and energetics of the Xe-substitution by a CN substituent of two neighboring anions (Figure 3). The calculated TS is located +45 kJ/mol above the minimum in which both Xe are bound by the electrophilic boron sites. The total reaction enthalpy is exothermic by −237 kJ/mol. Therefore, we can conclude that there will be always a thermochemical driving force for the elimination of an NG in a bulk solid upon its substitution with one of the substituents of the neighboring anion. Small alkali cations allow very close contact of neighboring anions in a solid. Additionally, alkali cations like Li^+^ and Na^+^ readily form complexes with water (alkali hydrates) and may, therefore, bind background water molecules, which could then attack [B_12_X_11_NG]^−^ adducts. Therefore, we conclude that alkali cations may be not the ideal choice to form a stable compound in the condensed phase.

Thus, the only possibility to isolate the desired NG-containing salt is the “installation” of strong kinetic barriers that should hinder the substitution of the NG, at least at sufficiently low temperatures. We consider that bulky, inert and non-hydrophilic cations should be much better suited than alkali cations since they may push the individual anions further apart from each other, which should affect substantial kinetic barriers for direct reactions between [B_12_X_11_NG]^−^ ions in the condensed phase.

The above considerations raise the question how large these barriers have to be and how effectively they can stabilize a B-NG bond. We consider the answer to this question in our case neither general nor straightforward. The well-characterized [F_5_C_6_Xe]^+^${\text{AsF}}_{6}^{-}$ salt (Frohn et al., 1998) can serve as an example of a NG compound, which is, though instable thermodynamically, very inert kinetically. The gas phase decomposition of this ion pair with the release of Xe and formation of C_6_F_6_ and AsF_5_ as reaction products is exothermic by −198 kJ/mol and we calculated a TS of +73 kJ/mol. In the bulk phase, this salt decomposes in the melt only above 120°C and is stable in a solvent like MeCN for 12 h without traces of decomposition (Frohn et al., 1998). However, these numbers may not be directly comparable to the enthalpy and TS calculated for our system because a variety of complex factors determine probabilities for reactive collisions between the reagents in the condensed phase. Reaction pathways, as calculated for the gas phase, are often not feasible in the solid state. Therefore, we cannot directly predict, based on calculated transition states, that the proposed [B_12_(CN)_11_Xe]^−^ salts may be stable at a certain temperature. Still, we would like to discuss our ideas regarding the stabilization of [B_12_(CN)_11_Xe]^−^ in the bulk phase, which may help to increase the temperature at which such salts may be stable.

It has been shown that different types of WCAs are optimally suited for the stabilization of [F_5_C_6_Xe]^+^ cation (Frohn and Bardin, 2001). Several WCAs, such as ${\text{AsF}}_{6}^{-}$ (Frohn et al., 1998) and [BY_4_]^−^ (Y = CF_3_, C_6_F_5_, CN, OTeF_5_) (Koppe et al., 2007, 2008), have been successfully tested affording compounds stable at temperatures up to 120°C ([F_5_C_6_Xe]^+^${\text{AsF}}_{6}^{-}$ salt). Even less F-substituted arylxenon derivatives (Aryl = 2,6-F_2_C_6_H_3_, 2-FC_6_H_4_, 4-FC_6_H_4_) were found to be stable at temperatures slightly below 0°C, when inert tetrafluoroborate [BF_4_]^−^ was used as a counterion (Naumann et al., 1993). In our case, we should look for bulky weakly coordinating cations [“weakly coordinating cations” is a rather recent concept complementary to the much older concept of “weakly coordinating anions”; see, for example (Price et al., 2009; Fischer et al., 2014; Moritz et al., 2015; Mann et al., 2018)], which do not contain any nucleophilic site that can attack the B-NG bond. Preferentially, we need to find a chemically inert, symmetric, bulky, and rigid cationic species that will be able to reliably separate [B_12_X_11_NG]^−^ anions from each other in the solid phase. The most suitable and easily accessible candidates would be either tetrahedral tetraphenylphosphonium derivatives (Fischer et al., 2014; Moritz et al., 2015) or stable metal complexes with a permanent positive charge. One of such candidates is the cobaltocenium cation [Cp_2_Co]^+^, a sandwich 18 *e* organometallic compound of high structural and chemical stability (Zhu et al., 2020). Cobaltocenium and its permethylated derivative [${\text{Cp}}_{2}^{*}$Co]^+^ (Cp^*^ denotes a permethylated cyclopentadienyl ligand) have been used as inert counterions for stabilization of labile anionic species, such as reactive radical anions (Morgan et al., 2011), in the solid state. Thus, [${\text{Cp}}_{2}^{*}$Co]^+^ seems to be a promising candidate also in our case: it is chemically inert, does not contain any nucleophilic site, and due to its large size it should keep the reactive anions much better separated from each other in a solid lattice than small alkali cations. Additionally, it possesses relatively high symmetry with a five-fold rotational axis, similar to the [B_12_(CN)_11_NG]^−^ adducts.

**Figure 4:**
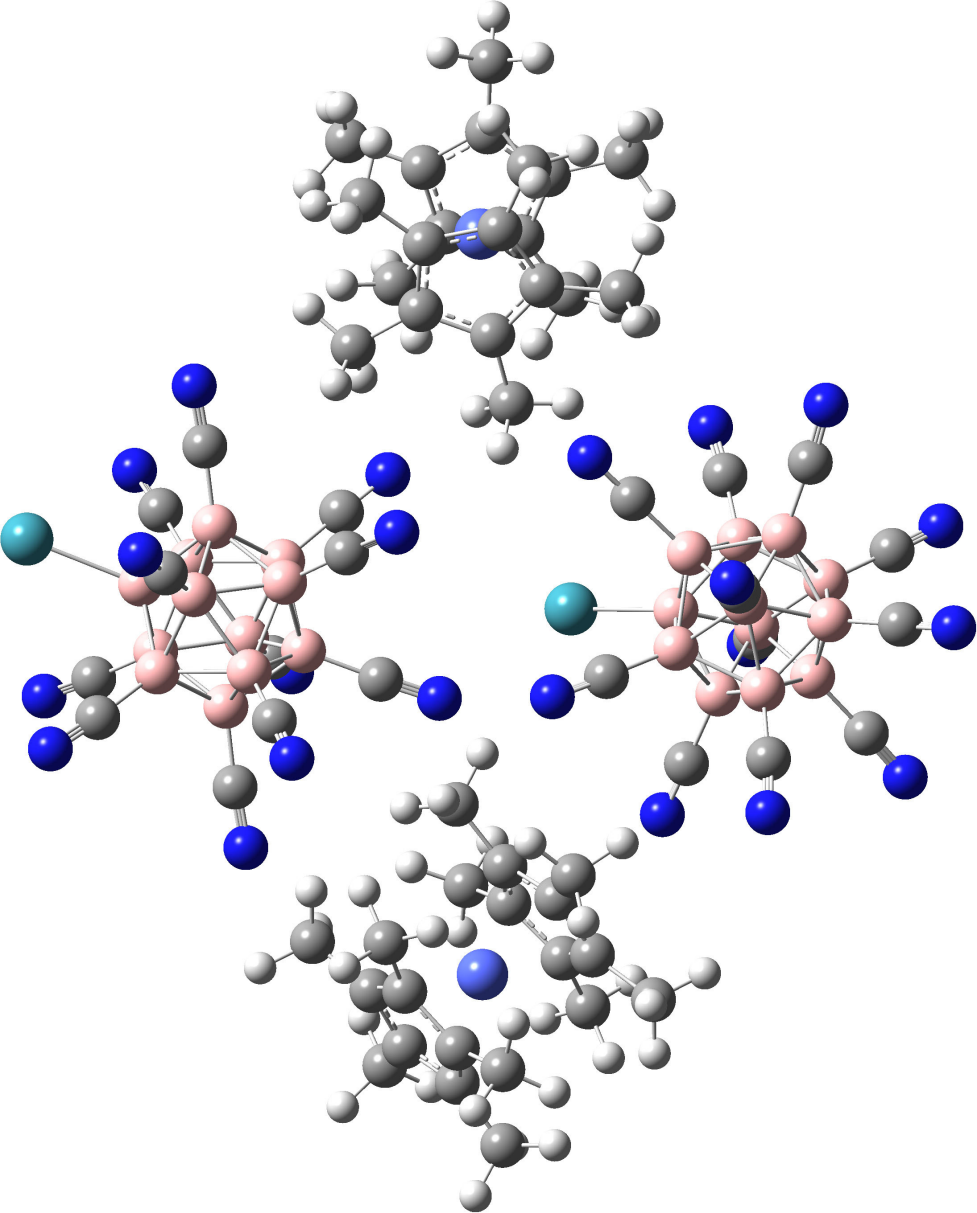
Optimized geometry of a fragment of the {(Co${\text{Cp}}_{2}^{*}$)·[B_12_(CN)_11_Xe]^−^} salt comprising an assembly of two anions and two cations. Calculation was performed on B3LYP-GD3BJ/6-311G (SDD) level of theory. We note that we optimized the structure of (Co${\text{Cp}}_{2}^{*}$)_2_[B_12_(CN)_11_Xe]_2_ also on B3LYP-GD3BJ/6-311++G(2d,2p) (SDD) level of theory resulting in a highly similar geometry to the one shown here (see Supplementary Material, section Substitution of Xe via insertion of [B_12_CN_11_]^−^ into a C-H bond of Co${\text{Cp}}_{2}^{*}$ for coordinates), but no frequency analysis was performed. Co is shown in pale blue. For other atom assignments see Figure 2.

A simple model based on two anions and two cations (see the optimized geometry in Figure 4) indeed demonstrates that [B_12_(CN)_11_Xe]^−^ anions are spatially better separated from each other than in the case of (Li[B_12_(CN)_11_Xe]^−^)_2_. The distance between the anions increases by roughly 1 Å in the optimized geometry of Figure 4 compared to Figure 3A. We expect that this “anion distancing” substantially increases the barrier for their intermolecular reaction with NG elimination. In addition, the outer surface of the cations represents rather chemically inert methyl groups, which have very low nucleophilicity. We calculated a barrier of 131 kJ/mol for the nucleophilic substitution of the Xe by the methyl group (see section Supplementary Material, section Substitution of Xe via Insertion of [B_12_CN_11_]^−^ into a C-H Bond of Co${\text{Cp}}_{2}^{*}$). The cyclopentadienyl π-systems binds to a positive metal and is not nucleophilic.

This molecular system between a [B_12_X_11_NG]^−^ anionic adducts and a weakly coordinating cation may constitute the first approach to the new generation of ionic noble gas compounds prepared by molecular ion deposition methods. It has been demonstrated that the soft-landing technique is well-suited for the preparation of thin layer materials based on *closo*-dodecaborate anions (Warneke et al., 2018) and can be also used for the isolation of the products of reactive fragment ions generated in the gas phase (Warneke et al., 2020). We plan to exploit the possibilities of these method for preparation of the bulk phases of the (Co${\text{Cp}}_{2}^{*}$)^+^·[B_12_X_11_Xe]^−^} salts, which may be classified as supersalts following the recently suggested definition (Giri et al., 2014).

## Conclusions

Recently developed anionic electrophiles based on *closo*-dodecaborate anions have opened up new possibilities for the formation and investigation of room temperature stable anionic NG derivatives and may allow the preparation of a new class of condensed phase NG compounds. Using explanations, which are intuitively understandable to chemist, we have summarized the concepts that explain how the vacant site of rigid anionic electrophiles possesses a unique chemical nature that facilitates binding of noble gases. Computational investigations indicate that the cyanated *closo*-dodecaborate derivative should form a xenon adduct likely isolable at ambient conditions. Since these NG adducts with anionic electrophiles can be generated only in the gas phase of a mass spectrometer, soft molecular ion deposition methods should be used for their isolation on a solid substrate. For the stabilization of the soft-landed ionic NG species we suggest using a weakly coordinating cation that should be co-deposited with it and serve as a counterion. Using computational methods, we have demonstrated that permethylated cobaltocenium cation may be a suitable candidate for the preparation of the bulk phase NG derivatives of anionic electrophiles. Currently, experimental soft-landing setups capable of performing these experiments are designed and may be used in future to prepare ionic NG-containing material layers that cannot be prepared using the “common” condensed phase synthetic methods.

## Methods

All calculations were performed with the Gaussian16 software, revision C.01 (Frisch et al., 2016), using hybrid functionals, which deliver reliable and consistent results in modeling of different types of *closo*-borate anions. If not stated otherwise, we employed DFT calculations on B3LYP-GD3BJ/6-311++G(2d,2p) (Krishnan et al., 1980; McLean and Chandler, 1980; Clark et al., 1983; Frisch et al., 1984; Lee et al., 1988; Miehlich et al., 1989; Becke, 1993) level of theory with SDD basis and pseudo potentials for Xe (Nicklass et al., 1995). All calculations included empirical dispersion corrections according to Grimme's D3 method (Grimme et al., 2010) with Becke-Johnson damping (GD3BJ) (Grimme et al., 2011). Subsequent frequency analyses were used to confirm that minima on the potential energy surface were obtained after optimization due to the absence of imaginary frequencies. Zero Kelvin reaction enthalpies were calculated by subtracting the Zero-Point Vibrational Energy (ZPVE) corrected electronic energies of the reaction educts from that of the reaction products. In case of attachment reactions, the Basis Set Superposition Error (BSSE) was corrected by performing a Counterpoise calculation (Boys and Bernardi, 1970; Simon et al., 1996) at the optimized geometry of the adduct. Only singlet states were considered due to the large singlet-triplet gap for [B_12_(CN)_11_]^−^ electrophilic anions (around 180 kJ/mol) and their adducts. All calculated energies are relative (most values herein have not been confirmed using high-level correlated methods); however, DFT values for NG adduct to electrophilic anion for the case of [B_12_(CN)_11_]^−^ and Ar have been shown to be sufficiently similar to CCSD(T) values (deviations <10%) and perfectly reproduce the general trends (Mayer et al., 2019). We additionally performed calculations on BMK-GD3BJ/aug-cc-pVTZ (SDD) level of theory (Dunning, 1989; Boese and Martin, 2004) on two example reaction discussed in this perspective to confirm the observed trends in thermochemistry as obtained from the standard B3LYP approach. Cartesian coordinates of all relevant structures are given in Supplementary Material, section Cartesian Coordinates of Relevant Species.

## Data Availability Statement

All datasets generated for this study are included in the article/Supplementary Material.

## Author Contributions

MR performed all computational investigations and co-wrote the manuscript. VA conceived some parts of the concept, performed literature search and analysis, and co-wrote the manuscript. JW initiated, designed, coordinated the study, and wrote the major part of the manuscript. All authors contributed to the article and approved the submitted version.

## Conflict of Interest

The authors declare that the research was conducted in the absence of any commercial or financial relationships that could be construed as a potential conflict of interest.
