# Supplementary material for: New Perspectives in the Noble Gas Chemistry Opened by Electrophilic Anions
Source: Front Chem. 2020 Nov 13;8:580295. doi: 10.3389/fchem.2020.580295 (PMC7691601; doi:10.3389/fchem.2020.580295)
Supplement: Supplementary file 1 [file Data_Sheet_1.docx]

Supplementary Material

**New Perspectives in the Noble Gas Chemistry Opened by Electrophilic Anions**

Markus Rohdenburg^1^, Vladimir A. Azov^2^, Jonas Warneke^3,4^

^1^ Institut für Angewandte und Physikalische Chemie, Fachbereich 2-Biologie/Chemie, Universität Bremen, 28359 Bremen, Germany

^2^ Department of Chemistry, University of the Free State, PO Box 339, 9300 Bloemfontein, South Africa

^3^ Wilhelm-Ostwald-Institut für Physikalische und Theoretische Chemie, Universität Leipzig, 04103 Leipzig, Germany

^4^ Leibniz Institute of Surface Engineering (IOM), Department Functional Surfaces, 04318 Leipzig, Germany

# Substitution of Xe by N_2_ in [H_3_CXe]^+^


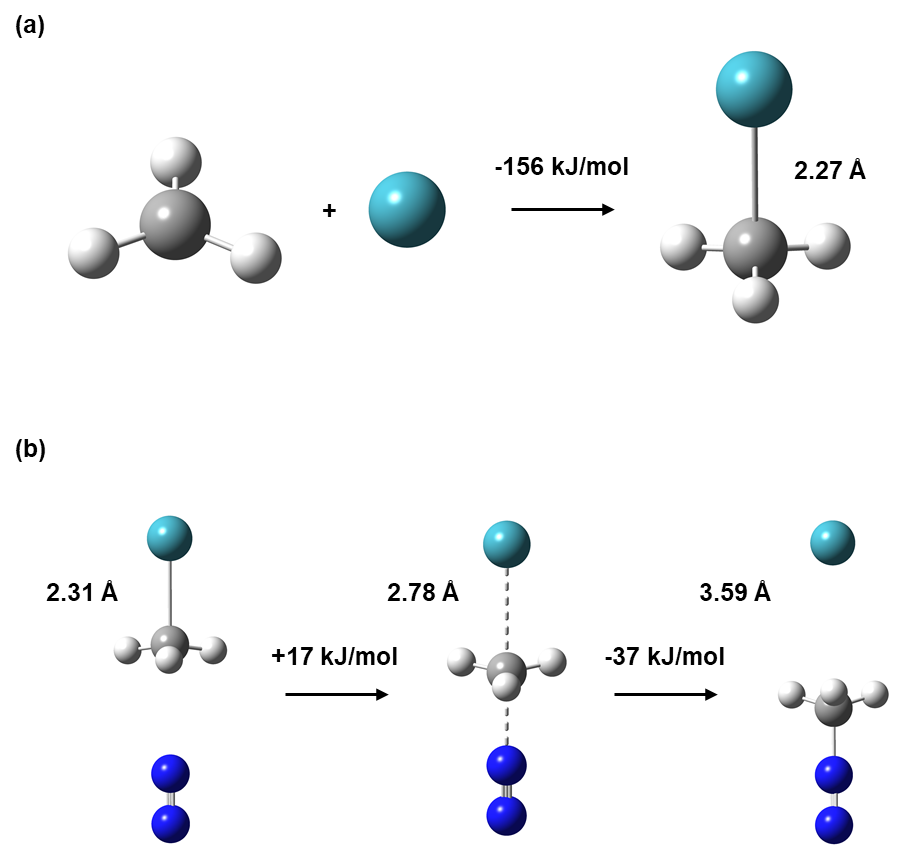


**Supplementary Figure 1.** (a) Attachment of Xe to CH_3_^+^ is calculated to be exothermic by
ΔH_0K_ = ‑156 kJ/mol. (b) Substitution of Xe in [H_3_CXe]^+^ via backside attack of N_2_ and a TS involving a planar CH_3_^+^ moiety. All C-Xe bond length are given in Å, all stated energies are ΔH_0K_. Calculations were done on B3LYP-GD3BJ/6-311++G(2d,2p) (SDD) level of theory.

# Substitution of Xe by N_2_ on BMK-GD3BJ/aug-cc-pVTZ(SDD) level of theory


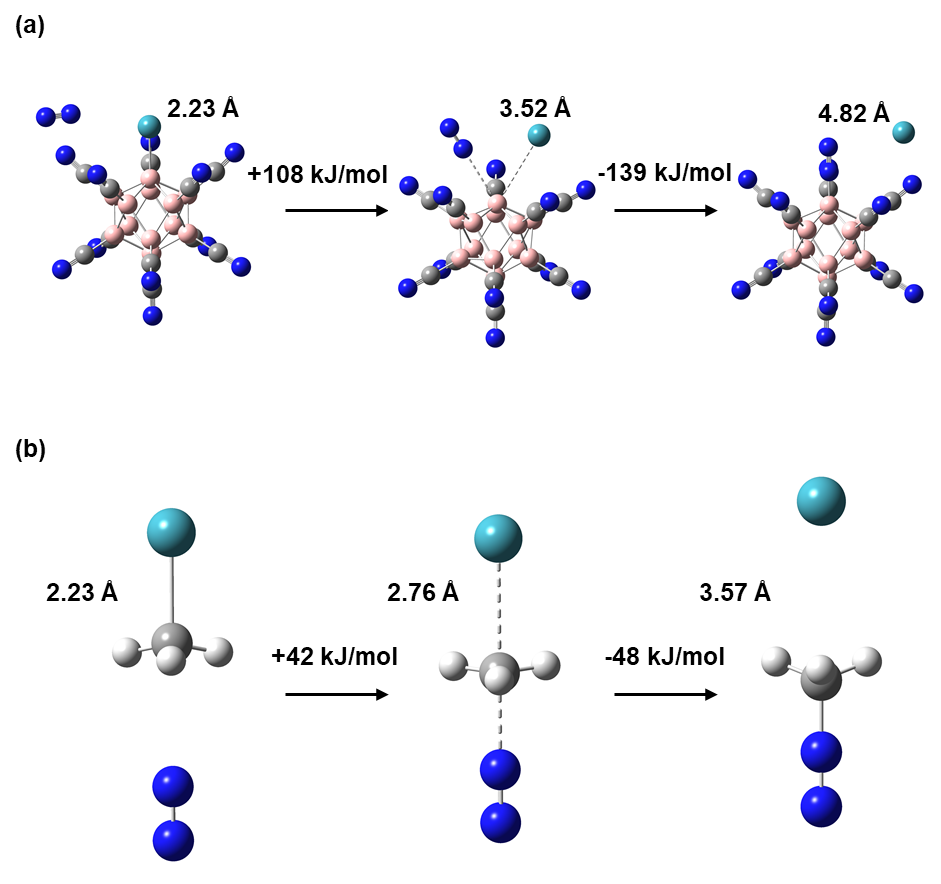


Supplementary Figure 2. Substitution of Xe by N_2_ in (a) [B_12_(CN)_11_Xe]^-^ and (b) [H_3_CXe]^+^ calculated on BMK-GD3BJ/aug-cc-pVTZ (SDD) level of theory. All B/C-Xe bond length are given in Å, all stated energies are ΔH_0K_.

# Substitution of Xe via insertion of [B_12_CN_11_]^-^ into a C-H bond of CoCp^*^_2_


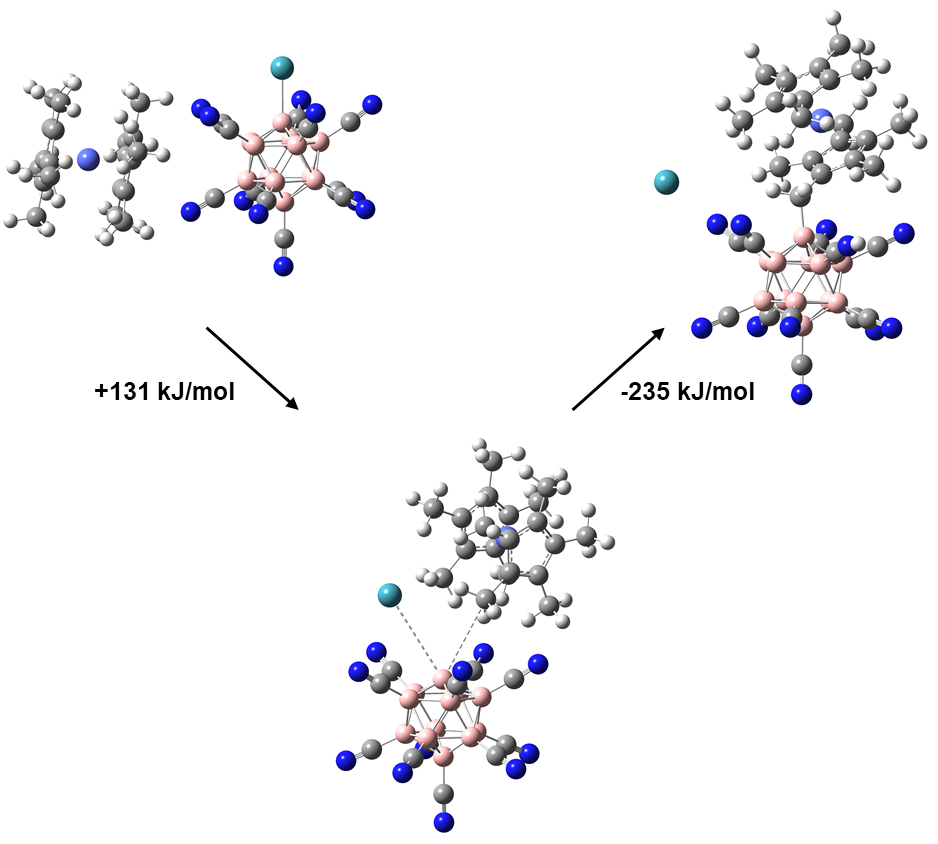


Supplementary Figure 3. Substitution of Xe in [B_12_(CN)_11_Xe] via insertion of the electrophilic boron site into a C-H bond of CoCp^*^_2_. All stated energies are ΔH_0K_.

# Cartesian Coordinates of Relevant Species

**Supplementary Table 1.** Cartesian Coordinates (in Å) of [B_12_(CN)_11_]^-^ on B3LYP-GD3BJ/6-311++G(2d,2p) (SDD) level of theory.

H_0K_ = ‑1320.298148 Ha

Atom X Y Z

B -1.277120000 -0.843814000 0.555263000

B 1.299590000 0.858592000 -0.962198000

B 0.000082000 0.000045000 1.474471000

B 0.407830000 -1.475329000 0.555111000

B -0.546770000 -1.458537000 -0.962156000

B -1.556114000 0.069252000 -0.962091000

B -1.197061000 0.953829000 0.555194000

B 0.537291000 1.433331000 0.555169000

B -0.415042000 1.501256000 -0.962147000

B -0.000059000 -0.000032000 -1.654740000

B 1.218107000 -0.970673000 -0.962264000

B 1.529214000 -0.068056000 0.555112000

C -2.269832000 1.808627000 1.223908000

C -2.875571000 0.128017000 -1.722197000

C -2.421545000 -1.599868000 1.224106000

C -1.010316000 -2.695326000 -1.722180000

C 0.773278000 -2.797356000 1.223951000

C 2.251102000 -1.793810000 -1.722217000

C 2.899447000 -0.129071000 1.223991000

C 2.401665000 1.586649000 -1.722147000

C 1.018683000 2.717707000 1.223956000

C -0.766913000 2.774278000 -1.722264000

C 0.000045000 0.000086000 3.002304000

N 3.021003000 -2.406927000 -2.318057000

N -1.355404000 -3.617093000 -2.317953000

N -3.289065000 -2.173310000 1.716653000

N -3.858799000 0.171415000 -2.318074000

N -1.029382000 3.722691000 -2.318345000

N 1.383404000 3.691692000 1.716259000

N 0.000000000 0.000292000 4.152654000

N 1.050686000 -3.799584000 1.716506000

N -3.083465000 2.456474000 1.716173000

N 3.938394000 -0.174860000 1.716454000

N 3.222625000 2.129362000 -2.318111000

**Supplementary Table 2.** Cartesian Coordinates (in Å) of [B_12_(CN)_11_-Xe]^-^ on B3LYP-GD3BJ/6-311++G(2d,2p) (SDD) level of theory.

H_0K_ = ‑1335.881428 Ha

Atom X Y Z

B -1.428439000 0.897501000 1.234095000

B 0.082160000 -0.900716000 -1.238119000

B -2.359644000 0.000037000 -0.000054000

B -1.428418000 1.451077000 -0.472254000

B 0.082146000 1.456387000 0.472434000

B 0.082173000 0.000829000 1.531093000

B -1.428426000 -0.896277000 1.234894000

B -1.428393000 -1.451449000 -0.470759000

B 0.082150000 -1.455887000 0.474044000

B 0.915116000 0.000065000 -0.000043000

B 0.082114000 0.899239000 -1.239190000

B -1.428434000 -0.000800000 -1.526017000

C -2.094151000 -1.704456000 2.348366000

C 0.869199000 0.001423000 2.841641000

C -2.094113000 1.706789000 2.346792000

C 0.869003000 2.703088000 0.876848000

C -2.093872000 2.759481000 -0.898143000

C 0.869121000 1.669065000 -2.299823000

C -2.093907000 -0.001535000 -2.901975000

C 0.869172000 -1.671926000 -2.297734000

C -2.093981000 -2.760239000 -0.895288000

C 0.869071000 -2.702099000 0.879770000

C -3.888462000 0.000074000 -0.000166000

N 1.541981000 2.217632000 -3.055469000

N 1.541567000 3.591486000 1.165067000

N -2.577946000 2.320896000 3.191015000

N 1.541860000 0.001876000 3.775552000

N 1.541701000 -3.590083000 1.169103000

N -2.577593000 -3.753340000 -1.217470000

N -5.038963000 0.000148000 -0.000483000

N -2.577360000 3.752300000 -1.221388000

N -2.578036000 -2.317684000 3.193197000

N -2.577328000 -0.002074000 -3.946120000

N 1.542038000 -2.221591000 -3.052573000

Xe 3.194140000 0.000093000 -0.000099000

**Supplementary Table 3.** Cartesian Coordinates (in Å) of [B_12_(CN)_11_-Xe···N_2_]^-^ on B3LYP-GD3BJ/6-311++G(2d,2p) (SDD) level of theory.

H_0K_ = ‑1445.443607 Ha

Atom X Y Z

B -1.449284000 0.791388000 1.451196000

B -0.342806000 -0.603956000 -1.456379000

B -2.484630000 0.660538000 0.000143000

B -1.095343000 1.784862000 0.000779000

B 0.231547000 1.006814000 0.900151000

B -0.343061000 -0.605277000 1.455783000

B -2.021787000 -0.815055000 0.896284000

B -2.021689000 -0.814207000 -0.897272000

B -0.698127000 -1.600992000 -0.000833000

B 0.603309000 -0.439778000 -0.000143000

B 0.231504000 1.007769000 -0.899237000

B -1.449106000 0.792689000 -1.450642000

C -3.022555000 -1.640397000 1.704616000

C 0.261135000 -1.249981000 2.703221000

C -1.933257000 1.415354000 2.759843000

C 1.323321000 1.748323000 1.671722000

C -1.258843000 3.304552000 0.001492000

C 1.323397000 1.749804000 -1.670135000

C -1.932991000 1.417948000 -2.758712000

C 0.261542000 -1.247543000 -2.704312000

C -3.022346000 -1.638811000 -1.706500000

C -0.398985000 -3.100087000 -0.001397000

C -3.924686000 1.173967000 0.000378000

N 2.204119000 2.247754000 -2.218549000

N 2.204191000 2.245775000 2.220347000

N -2.280130000 1.881921000 3.752781000

N 0.797151000 -1.745829000 3.592882000

N -0.081457000 -4.206356000 -0.001774000

N -3.761481000 -2.271638000 -2.320620000

N -5.008372000 1.560334000 0.000586000

N -1.361483000 4.450590000 0.002044000

N -3.761783000 -2.273782000 2.318051000

N -2.279819000 1.885522000 -3.751192000

N 0.797682000 -1.742605000 -3.594335000

Xe 2.743926000 -1.208214000 -0.000081000

N 4.957852000 2.931308000 0.000507000

N 4.712239000 1.868547000 -0.000164000

**Supplementary Table 4.** Cartesian Coordinates (in Å) of TS [B_12_(CN)_11_···Xe···N_2_]^-^ on B3LYP-GD3BJ/6-311++G(2d,2p) (SDD) level of theory.

H_0K_ = ‑1445.409259 Ha

Atom X Y Z

B 1.651098000 -0.115182000 -1.578681000

B 0.353385000 0.038888000 1.595903000

B 2.613526000 -0.520599000 -0.129892000

B 2.132904000 1.169759000 -0.423708000

B 0.483419000 1.162872000 -1.124207000

B -0.052504000 -0.576363000 -1.258511000

B 1.284657000 -1.595797000 -0.635555000

B 1.533109000 -1.222452000 1.104644000

B -0.120409000 -1.277395000 0.425644000

B -0.423656000 0.389628000 0.109821000

B 0.744421000 1.539632000 0.640195000

B 2.056037000 0.485085000 1.235252000

C 1.515841000 -2.993673000 -1.203924000

C -1.035455000 -1.011949000 -2.339250000

C 2.215221000 -0.180397000 -2.995903000

C -0.052731000 2.217901000 -2.085308000

C 3.127426000 2.264660000 -0.801915000

C 0.481149000 2.926065000 1.216043000

C 2.984717000 0.961171000 2.349626000

C -0.310498000 0.135853000 2.965278000

C 1.989913000 -2.285249000 2.100798000

C -1.105094000 -2.371878000 0.818635000

C 4.071901000 -0.961733000 -0.245517000

N 0.278747000 3.970641000 1.653194000

N -0.487261000 3.013744000 -2.793318000

N 2.627984000 -0.225330000 -4.068998000

N -1.796107000 -1.329799000 -3.141634000

N -1.829057000 -3.212688000 1.122041000

N 2.324028000 -3.084649000 2.857932000

N 5.169837000 -1.293766000 -0.333031000

N 3.866527000 3.098546000 -1.088761000

N 1.676910000 -4.048037000 -1.635567000

N 3.674661000 1.324150000 3.195867000

N -0.848203000 0.220235000 3.978920000

Xe -3.903568000 -0.378876000 0.030756000

N -2.931296000 3.419935000 0.097670000

N -2.267004000 2.556201000 0.136151000

**Supplementary Table 5.** Cartesian Coordinates (in Å) of [B_12_(CN)_11_-N_2_···Xe]^-^ on B3LYP-GD3BJ/6-311++G(2d,2p) (SDD) level of theory.

H_0K_ = ‑1445.463279 Ha

B -2.594324000 0.599083000 0.897408000

B 0.022087000 -0.649855000 -0.901673000

B -2.700525000 -0.944074000 -0.000146000

B -2.594389000 0.599460000 -0.897057000

B -1.495659000 1.672898000 0.000366000

B -0.915446000 0.785614000 1.458885000

B -1.661652000 -0.829658000 1.451026000

B -1.085404000 -1.711842000 -0.000367000

B 0.022156000 -0.650234000 0.901301000

B 0.064396000 0.859193000 0.000135000

B -0.915558000 0.786229000 -1.458580000

B -1.661756000 -0.829046000 -1.451350000

C -1.987243000 -1.550691000 2.758173000

C -0.478729000 1.553292000 2.705419000

C -3.760403000 1.165822000 1.705849000

C -1.553889000 3.199510000 0.000682000

C -3.760522000 1.166548000 -1.705175000

C -0.478913000 1.554442000 -2.704808000

C -1.987452000 -1.549524000 -2.758777000

C 1.249439000 -1.121859000 -1.679190000

C -0.890857000 -3.227384000 -0.000703000

C 1.249538000 -1.122590000 1.678555000

C -3.979594000 -1.779825000 -0.000278000

N -0.083644000 2.159054000 -3.600781000

N -1.514726000 4.349740000 0.000921000

N -4.629516000 1.605039000 2.318718000

N -0.083422000 2.157523000 3.601630000

N 2.206889000 -1.422336000 2.242305000

N -0.726938000 -4.366241000 -0.000962000

N -4.942695000 -2.409349000 -0.000379000

N -4.629675000 1.606034000 -2.317796000

N -2.219163000 -2.087624000 3.749021000

N -2.219452000 -2.086031000 -3.749836000

N 2.206772000 -1.421337000 -2.243114000

Xe 4.957791000 -0.053600000 -0.000004000

N 2.204791000 2.321553000 0.000320000

N 1.306693000 1.698130000 0.000242000

**Supplementary Table 6.** Cartesian Coordinates (in Å) of CH_3_^+^ on B3LYP-GD3BJ/6-311++G(2d,2p) (SDD) level of theory.

H_0K_ = ‑39.463752 Ha

Atom X Y Z

C 0.000000000 0.000000000 -0.000016000

H 0.000000000 1.090666000 0.000031000

H 0.944545000 -0.545333000 0.000031000

H -0.944545000 -0.545333000 0.000031000

**Supplementary Table 7.** Cartesian Coordinates (in Å) of CH_3_-Xe^+^ on B3LYP-GD3BJ/6-311++G(2d,2p) (SDD) level of theory.

H_0K_ = ‑55.062821 Ha

Atom X Y Z

C 0.000000000 0.000000000 -1.935240000

H 0.000000000 1.060891000 -2.147527000

H 0.918758000 -0.530445000 -2.147527000

H -0.918758000 -0.530445000 -2.147527000

Xe 0.000000000 0.000000000 0.334334000

**Supplementary Table 8.** Cartesian Coordinates (in Å) of CH_3_-Xe···N_2_^+^ on B3LYP-GD3BJ/6-311++G(2d,2p) (SDD) level of theory.

H_0K_ = ‑164.626222 Ha

Atom X Y Z

C -1.002825000 0.001459000 0.000755000

H -1.195864000 -0.731292000 0.770702000

H -1.194796000 1.034832000 0.250427000

H -1.196145000 -0.298839000 -1.018775000

Xe 1.305731000 -0.000159000 -0.000082000

N -3.804932000 0.000073000 0.000063000

N -4.895883000 -0.000769000 -0.000416000

**Supplementary Table 9.** Cartesian Coordinates (in Å) of TS CH_3_···Xe···N_2_^+^ on B3LYP-GD3BJ/6-311++G(2d,2p) (SDD) level of theory.

H_0K_ = ‑164.619882 Ha

C 1.483435000 0.000163000 0.000413000

H 1.444217000 -1.019218000 -0.349666000

H 1.444261000 0.813053000 -0.707338000

H 1.444270000 0.206672000 1.058276000

Xe -1.296391000 -0.000016000 -0.000040000

N 3.509995000 0.000050000 0.000153000

N 4.600257000 -0.000142000 -0.000377000

**Supplementary Table 10.** Cartesian Coordinates (in Å) of CH_3_-N_2_···Xe^+^ on B3LYP-GD3BJ/6-311++G(2d,2p) (SDD) level of theory.

H_0K_ = ‑164.633994 Ha

Atom X Y Z

C -2.166526000 -0.000233000 0.000764000

H -1.859357000 0.909116000 -0.513247000

H -1.859679000 -0.900366000 -0.529408000

H -1.860420000 -0.009619000 1.045617000

Xe 1.425514000 0.000016000 -0.000057000

N -3.625778000 0.000043000 -0.000020000

N -4.716953000 0.000155000 -0.000615000

**Supplementary Table 11.** Cartesian Coordinates (in Å) of B_5_O_7_^+^ on B3LYP-GD3BJ/6-311++G(2d,2p) (SDD) level of theory.

H_0K_ = ‑651.205044 Ha

Atom X Y Z

O -1.224856000 -2.064211000 0.000117000

B -1.238534000 -0.586926000 0.000029000

O 0.000001000 0.001880000 0.000009000

B 1.238537000 -0.586924000 0.000051000

O 1.224861000 -2.064210000 0.000105000

B 0.000003000 -2.403384000 0.000127000

O 2.415142000 -0.002469000 -0.000001000

B 2.863492000 1.265332000 -0.000085000

O 3.268261000 2.392800000 -0.000149000

O -2.415140000 -0.002472000 0.000002000

B -2.863490000 1.265329000 -0.000059000

O -3.268273000 2.392791000 -0.000122000

**Supplementary Table 12.** Cartesian Coordinates (in Å) of B_5_O_7_-Xe^+^ on B3LYP-GD3BJ/6-311++G(2d,2p) (SDD) level of theory.

H_0K_ = ‑666.789459 Ha

O -0.080661000 -1.198801000 -0.000011000

B 1.342173000 -1.211008000 0.000103000

O 1.979534000 0.000000000 0.000146000

B 1.342173000 1.211008000 0.000087000

O -0.080661000 1.198801000 -0.000013000

B -0.615985000 0.000000000 -0.000052000

O 1.948592000 2.396885000 0.000150000

B 3.228695000 2.785022000 0.000263000

O 4.370225000 3.157081000 0.000364000

O 1.948592000 -2.396885000 0.000148000

B 3.228695000 -2.785022000 0.000243000

O 4.370225000 -3.157081000 0.000330000

Xe -2.931028000 0.000000000 -0.000225000

**Supplementary Table 13.** Cartesian Coordinates (in Å) of B_5_O_7_-Xe-[HCB_11_Cl_11_] on B3LYP-GD3BJ/6-311++G(2d,2p) (SDD) level of theory.

H_0K_ = ‑6042.445560 Ha

Atom X Y Z

B 1.132448000 0.957115000 0.000026000

B 0.818546000 -0.767234000 -0.000521000

B 1.471965000 -0.027975000 -1.464820000

B 2.648324000 1.205605000 -0.903055000

B 2.647808000 1.205057000 0.904180000

B 1.471143000 -0.028870000 1.464606000

B 2.047928000 -1.619440000 0.904169000

B 3.210235000 -0.390709000 1.454448000

B 3.930348000 0.367542000 0.000684000

B 3.211076000 -0.389844000 -1.453959000

B 2.048480000 -1.618866000 -0.904965000

Cl -0.158771000 2.210005000 0.000126000

Cl -0.892393000 -1.588376000 -0.001400000

Cl 0.512504000 0.128517000 -2.954260000

Cl 2.957087000 2.679317000 -1.839624000

Cl 2.956006000 2.678176000 1.841868000

Cl 0.510905000 0.126768000 2.953627000

Cl 1.797107000 -3.142658000 1.771931000

Cl 4.186895000 -0.697073000 2.893581000

Cl 5.636606000 0.822110000 0.001218000

Cl 4.188582000 -0.695489000 -2.892654000

Cl 1.797972000 -3.141542000 -1.773821000

C 3.447165000 -1.278324000 0.000082000

H 4.228277000 -2.021413000 -0.000024000

O -2.489942000 0.292216000 1.192668000

B -3.242385000 1.454970000 1.193777000

O -3.613022000 2.019504000 0.000076000

B -3.242049000 1.456132000 -1.194121000

O -2.489805000 0.293282000 -1.194077000

B -2.077935000 -0.146518000 -0.000857000

O -3.605261000 1.982226000 -2.385232000

B -4.305918000 3.055072000 -2.724233000

O -4.946518000 4.012559000 -3.080350000

O -3.606060000 1.979553000 2.385403000

B -4.307057000 3.051682000 2.725903000

O -4.947948000 4.008458000 3.083414000

Xe -4.740928000 -2.505287000 -0.000594000

**Supplementary Table 14.** Cartesian Coordinates (in Å) of B_5_O_7_-[B_12_CN_11_]-Xe on B3LYP-GD3BJ/6-311++G(2d,2p) (SDD) level of theory.

H_0K_ = ‑1987.322249 Ha

Atom X Y Z

B 0.779975000 0.319592000 -1.481565000

B 2.321580000 -0.330612000 1.503424000

B -0.076644000 -0.014832000 0.029252000

B 0.798385000 -1.336476000 -0.757530000

B 2.289095000 -0.618368000 -1.408963000

B 2.284229000 1.149201000 -1.021655000

B 0.790035000 1.520176000 -0.132248000

B 0.812277000 0.607065000 1.425382000

B 2.304595000 1.326900000 0.778312000

B 3.140513000 0.003555000 -0.009450000

B 2.313679000 -1.532817000 0.151741000

B 0.818023000 -1.158958000 1.038765000

C 0.015533000 2.828409000 -0.254200000

C 3.037200000 2.136672000 -1.905549000

C -0.013468000 0.595010000 -2.755223000

C 3.045838000 -1.141383000 -2.624344000

C 0.029614000 -2.475821000 -1.418831000

C 3.092769000 -2.837373000 0.272032000

C 0.066642000 -2.145963000 1.926327000

C 3.106836000 -0.607492000 2.780174000

C 0.051667000 1.128424000 2.640612000

C 3.075668000 2.465886000 1.435180000

C -1.607302000 -0.018849000 0.037517000

N 3.751082000 -3.777305000 0.357592000

N 3.687375000 -1.517702000 -3.502407000

N -0.690820000 0.790084000 -3.664814000

N 3.676248000 2.849139000 -2.544442000

N 3.728052000 3.287461000 1.908022000

N -0.597649000 1.500228000 3.514873000

N -2.752153000 -0.018244000 0.036324000

N -0.619774000 -3.295926000 -1.898289000

N -0.635590000 3.773083000 -0.342396000

N -0.570791000 -2.855590000 2.569961000

N 3.769222000 -0.806423000 3.699875000

Xe 5.403641000 0.016146000 -0.035912000

O -4.849068000 1.185112000 0.016702000

B -6.236656000 1.196704000 0.001853000

O -6.915953000 0.004885000 -0.005261000

B -6.250456000 -1.194573000 0.002777000

O -4.862721000 -1.199287000 0.018158000

B -4.233726000 -0.010642000 0.023363000

O -6.893015000 -2.380905000 -0.003567000

B -8.176655000 -2.719667000 -0.018717000

O -9.330195000 -3.067288000 -0.032121000

O -6.865455000 2.390316000 -0.004846000

B -8.144944000 2.744375000 -0.018638000

O -9.294284000 3.105701000 -0.030752000

**Supplementary Table 15.** Cartesian Coordinates (in Å) of B_5_O_7_-Xe-[B_12_CN_11_] on B3LYP-GD3BJ/6-311++G(2d,2p) (SDD) level of theory.

H_0K_ = ‑1987.345844 Ha

Atom X Y Z

B -3.401574000 -0.982139000 -0.169833000

B -1.077738000 1.433767000 0.281188000

B -3.540582000 0.246071000 1.139708000

B -2.276832000 -1.022124000 1.217445000

B -1.663739000 -1.306296000 -0.457660000

B -2.574558000 -0.218111000 -1.552248000

B -3.727117000 0.741787000 -0.572638000

B -2.791200000 1.774347000 0.563008000

B -2.195423000 1.477326000 -1.094755000

B -0.961774000 0.223151000 -1.023874000

B -0.783650000 -0.274586000 0.682657000

B -1.899165000 0.687151000 1.671377000

C -5.058974000 1.202202000 -1.152605000

C -2.770795000 -0.593464000 -3.017698000

C -4.441657000 -2.078157000 -0.370289000

C -1.022182000 -2.616823000 -0.906932000

C -2.219799000 -2.135035000 2.259008000

C 0.630074000 -0.497915000 1.211929000

C -1.461381000 1.066933000 3.083582000

C 0.180736000 2.309077000 0.344077000

C -3.239288000 3.161407000 1.009410000

C -1.900063000 2.531767000 -2.161606000

C -4.705570000 0.249663000 2.122560000

N 1.761341000 -0.421294000 1.447067000

N -0.422591000 -3.542675000 -1.235446000

N -5.209522000 -2.920170000 -0.528735000

N -2.832303000 -0.859256000 -4.135639000

N -1.448059000 3.233452000 -2.955409000

N -3.530270000 4.224637000 1.339333000

N -5.573827000 0.254194000 2.877320000

N -2.094305000 -2.970288000 3.040436000

N -6.052560000 1.562484000 -1.607486000

N -1.017484000 1.338052000 4.110237000

N 1.267039000 2.686707000 0.265378000

O 0.136493000 0.314407000 -1.983666000

B 1.156141000 0.966144000 -2.263991000

O 2.314467000 1.517208000 -2.413226000

B 3.146299000 1.278550000 -1.284547000

O 4.102483000 2.094043000 -0.823335000

O 2.776295000 0.144529000 -0.603142000

B 4.463442000 1.963902000 0.515515000

B 2.894772000 0.242964000 0.755399000

O 3.806832000 1.038203000 1.323677000

O 5.434242000 2.715807000 1.058574000

B 6.226674000 3.667307000 0.572753000

O 6.967296000 4.521988000 0.160172000

Xe 3.200860000 -3.462303000 -0.027740000

**Supplementary Table 16.** Cartesian Coordinates (in Å) of Li[B_12_CN_11_] on B3LYP-GD3BJ/6-311++G(2d,2p) (SDD) level of theory.

H_0K_ = ‑1327.748285 Ha

Atom X Y Z

B -1.203712000 0.903067000 0.082461000

B 1.668458000 -0.919945000 0.442759000

B -0.440225000 -0.000002000 -1.264248000

B 0.340027000 1.465165000 -0.629024000

B 0.072375000 1.494422000 1.152654000

B -0.914794000 0.000045000 1.594233000

B -1.203762000 -0.902996000 0.082470000

B 0.339949000 -1.465191000 -0.628990000

B 0.072287000 -1.494396000 1.152687000

B 0.792145000 -0.000002000 1.587489000

B 1.668511000 0.919867000 0.442737000

B 1.311630000 -0.000045000 -1.062598000

C -2.571881000 -1.499629000 -0.276578000

C -1.866639000 0.000089000 2.778976000

C -2.571800000 1.499759000 -0.276605000

C -0.013661000 2.752259000 2.001305000

C 0.401682000 2.754921000 -1.435630000

C 2.947572000 1.697832000 0.703675000

C 2.272860000 -0.000082000 -2.242287000

C 2.947477000 -1.697974000 0.703713000

C 0.401532000 -2.754967000 -1.435571000

C -0.013823000 -2.752208000 2.001367000

C -1.303339000 0.000010000 -2.534876000

N 3.912823000 2.285575000 0.919133000

N -0.094673000 3.699113000 2.649853000

N -3.609744000 1.643399000 -0.753265000

N -2.609380000 0.000126000 3.657692000

N -0.094891000 -3.699040000 2.649940000

N 0.404297000 -3.724126000 -2.056009000

N -2.215095000 -0.000025000 -3.237395000

N 0.404501000 3.724069000 -2.056084000

N -3.609861000 -1.643238000 -0.753170000

N 2.982765000 -0.000111000 -3.147914000

N 3.912697000 -2.285765000 0.919182000

Li -3.992798000 0.000054000 -2.077277000

**Supplementary Table 17.** Cartesian Coordinates (in Å) of Li[B_12_CN_11_-Xe] on B3LYP-GD3BJ/6-311++G(2d,2p) (SDD) level of theory.

H_0K_ = ‑1343.341265 Ha

Atom X Y Z

B -1.460668000 -0.938194000 0.899373000

B 0.381864000 1.259326000 -0.903041000

B -2.191855000 0.429576000 -0.000006000

B -1.225523000 0.740362000 1.460496000

B 0.125960000 -0.436591000 1.466884000

B -0.032850000 -1.486196000 -0.000003000

B -1.460667000 -0.938191000 -0.899385000

B -1.225520000 0.740364000 -1.460503000

B 0.125962000 -0.436592000 -1.466891000

B 1.026846000 -0.098630000 -0.000002000

B 0.381862000 1.259325000 0.903036000

B -1.067903000 1.788358000 -0.000004000

C -2.500659000 -1.897688000 -1.498214000

C 0.481817000 -2.921422000 -0.000001000

C -2.500653000 -1.897686000 1.498218000

C 0.818771000 -0.968325000 2.716727000

C -1.870585000 1.225847000 2.754069000

C 1.319170000 2.183698000 1.672716000

C -1.540553000 3.237433000 -0.000005000

C 1.319174000 2.183697000 -1.672719000

C -1.870582000 1.225849000 -2.754077000

C 0.818773000 -0.968325000 -2.716732000

C -3.728556000 0.386107000 -0.000002000

N 2.099231000 2.823174000 2.226552000

N 1.402811000 -1.383169000 3.617318000

N -3.458802000 -2.518845000 1.646024000

N 0.911494000 -3.988999000 0.000008000

N 1.402810000 -1.383182000 -3.617320000

N -2.384758000 1.561010000 -3.727340000

N -4.811396000 -0.004944000 0.000001000

N -2.384760000 1.561010000 3.727332000

N -3.458802000 -2.518863000 -1.646002000

N -1.911980000 4.326384000 0.000008000

N 2.099240000 2.823178000 -2.226544000

Xe 3.266723000 -0.423323000 0.000002000

Li -4.767638000 -2.122514000 -0.000009000

**Supplementary Table 18.** Cartesian Coordinates (in Å) of [B_12_CN_11_]-[B_12_CN_11_-Xe]^2-^ on B3LYP-GD3BJ/6-311++G(2d,2p) (SDD) level of theory.

H_0K_ = ‑2656.256292 Ha

Atom X Y Z

B 2.244758000 -0.251222000 -1.512425000

B 3.757824000 0.252880000 1.511723000

B 1.345101000 0.002671000 0.003552000

B 2.245109000 -1.517023000 -0.224957000

B 3.753007000 -1.092428000 -1.073934000

B 3.754558000 0.684321000 -1.370818000

B 2.247543000 1.364137000 -0.706443000

B 2.249459000 1.096887000 1.078933000

B 3.757503000 1.515749000 0.227194000

B 4.586098000 -0.000659000 -0.000706000

B 3.755009000 -1.359027000 0.707713000

B 2.248090000 -0.683696000 1.376628000

C 1.592447000 2.585801000 -1.340396000

C 4.548431000 1.266387000 -2.539539000

C 1.587388000 -0.477268000 -2.868972000

C 4.545721000 -2.023855000 -1.989904000

C 1.588346000 -2.877551000 -0.427619000

C 4.549230000 -2.517640000 1.309229000

C 1.593561000 -1.296975000 2.609099000

C 4.554398000 0.467503000 2.797951000

C 1.595919000 2.078692000 2.044582000

C 4.553630000 2.805782000 0.419354000

C -0.191377000 0.003091000 0.004470000

N 5.240262000 -3.334438000 1.733246000

N 5.235854000 -2.680472000 -2.635696000

N 1.134814000 -0.650989000 -3.912400000

N 5.239303000 1.676714000 -3.363434000

N 5.245848000 3.715164000 0.554747000

N 1.145617000 2.833460000 2.787079000

N -1.333793000 0.002285000 0.003957000

N 1.136515000 -3.924143000 -0.583297000

N 1.141397000 3.525357000 -1.827773000

N 1.142795000 -1.768365000 3.556911000

N 5.246985000 0.618874000 3.704529000

Xe 6.863112000 -0.003121000 -0.003767000

B -5.214682000 -0.681027000 1.360040000

B -3.700817000 0.682250000 -1.363563000

B -6.146881000 -0.001313000 -0.002915000

B -5.215513000 1.083904000 1.066373000

B -3.704946000 0.250680000 1.505609000

B -3.702687000 -1.353223000 0.704027000

B -5.211665000 -1.505708000 -0.227837000

B -5.210527000 -0.250444000 -1.502828000

B -3.700032000 -1.086555000 -1.069232000

B -2.808963000 0.001101000 0.002390000

B -3.703841000 1.508815000 0.227740000

B -5.212885000 1.350021000 -0.702957000

C -5.893603000 -2.861200000 -0.432669000

C -2.953162000 -2.535452000 1.319518000

C -5.899429000 -1.294095000 2.584635000

C -2.956992000 0.469970000 2.821207000

C -5.901028000 2.059802000 2.026599000

C -2.955342000 2.827243000 0.427213000

C -5.896132000 2.565465000 -1.335598000

C -2.949063000 1.278239000 -2.554328000

C -5.891549000 -0.475947000 -2.855496000

C -2.947736000 -2.035710000 -2.002728000

C -7.678401000 -0.002489000 -0.005347000

N -2.351010000 3.795688000 0.573812000

N -2.352621000 0.630999000 3.787331000

N -6.413296000 -1.755066000 3.505375000

N -2.348102000 -3.403763000 1.771760000

N -2.340428000 -2.732716000 -2.688258000

N -6.402563000 -0.645571000 -3.872555000

N -8.829195000 -0.003481000 -0.007283000

N -6.415544000 2.793524000 2.748570000

N -6.405316000 -3.880383000 -0.586720000

N -6.408886000 3.479287000 -1.811340000

N -2.341879000 1.715798000 -3.428662000

**Supplementary Table 19.** Cartesian Coordinates (in Å) of Li_2_[B_12_CN_11_-Xe]_2_ on B3LYP-GD3BJ/6-311++G(2d,2p) (SDD) level of theory.

H_0K_ = ‑2686.726152 Ha

Atom X Y Z

B 6.457259000 0.208914000 0.896899000

B 3.590580000 0.618393000 -0.890781000

B 6.106441000 1.718674000 -0.000035000

B 6.457207000 0.208856000 -0.896893000

B 5.714369000 -1.144049000 0.000068000

B 4.900081000 -0.471646000 1.449927000

B 5.148257000 1.296135000 1.443867000

B 4.335508000 1.969270000 0.000008000

B 3.590633000 0.618449000 0.890931000

B 3.982264000 -0.836231000 0.000111000

B 4.899995000 -0.471739000 -1.449785000

B 5.148172000 1.296044000 -1.443854000

C 5.163441000 2.068444000 2.761561000

C 4.665249000 -1.292838000 2.715980000

C 7.725759000 0.008261000 1.718891000

C 6.222844000 -2.582949000 0.000098000

C 7.725658000 0.008151000 -1.718947000

C 4.665083000 -1.293009000 -2.715772000

C 5.163283000 2.068264000 -2.761601000

C 2.388776000 0.849571000 -1.815424000

C 3.645323000 3.331279000 -0.000004000

C 2.388881000 0.849700000 1.815621000

C 7.062535000 2.907057000 -0.000101000

N 4.372677000 -1.929296000 -3.629172000

N 6.501973000 -3.699389000 0.000124000

N 8.671247000 -0.156345000 2.353438000

N 4.372910000 -1.929071000 3.629437000

N 1.577977000 1.069915000 2.595467000

N 3.055888000 4.319606000 0.000006000

N 7.770971000 3.813487000 -0.000152000

N 8.671107000 -0.156498000 -2.353542000

N 5.081297000 2.626413000 3.764497000

N 5.081091000 2.626161000 -3.764573000

N 1.577845000 1.069700000 -2.595266000

Xe 2.688409000 -2.697956000 0.000204000

B -3.369373000 -1.303887000 0.897351000

B -4.653577000 1.307460000 -0.901521000

B -2.349714000 -0.155795000 -0.000125000

B -3.369425000 -1.303980000 -0.897423000

B -4.899435000 -1.465593000 0.000018000

B -4.806253000 -0.407781000 1.460766000

B -3.228466000 0.396653000 1.435372000

B -3.124713000 1.452496000 -0.000182000

B -4.653524000 1.307554000 0.901260000

B -5.603979000 0.143007000 -0.000044000

B -4.806339000 -0.407932000 -1.460846000

B -3.228554000 0.396502000 -1.435625000

C -2.381444000 0.775413000 2.658890000

C -5.591984000 -0.734238000 2.725351000

C -2.716490000 -2.425019000 1.700135000

C -5.789310000 -2.702928000 0.000110000

C -2.716583000 -2.425199000 -1.700118000

C -5.592150000 -0.734517000 -2.725348000

C -2.381589000 0.775187000 -2.659204000

C -5.301553000 2.439325000 -1.690097000

C -2.260921000 2.709444000 -0.000310000

C -5.301461000 2.439496000 1.689757000

C -0.826879000 -0.295619000 -0.000165000

N -6.237074000 -0.951828000 -3.653300000

N -6.530896000 -3.582846000 0.000180000

N -2.157963000 -3.229880000 2.303711000

N -6.236846000 -0.951454000 3.653368000

N -5.843322000 3.268116000 2.276303000

N -1.557362000 3.620149000 -0.000444000

N 0.316330000 -0.405681000 -0.000228000

N -2.158076000 -3.230131000 -2.303618000

N -1.617487000 1.072821000 3.461477000

N -1.617580000 1.072580000 -3.461747000

N -5.843443000 3.267887000 -2.276698000

Xe -7.856837000 0.339067000 0.000011000

Li 0.235055000 1.473365000 -3.892249000

Li 0.235081000 1.473477000 3.892375000

**Supplementary Table 20.** Cartesian Coordinates (in Å) of Li_2_[B_12_CN_11_···Xe]-[B_12_CN_11_-Xe] on B3LYP-GD3BJ/6-311++G(2d,2p) (SDD) level of theory.

H_0K_ = ‑2686.816541 Ha

Atom X Y Z

B -5.445246000 1.185742000 0.895286000

B -3.975933000 -1.319910000 -0.880320000

B -6.405490000 -0.041055000 -0.000076000

B -5.445132000 1.185592000 -0.895556000

B -3.918642000 1.460281000 -0.000073000

B -3.951939000 0.398121000 1.429664000

B -5.475204000 -0.527011000 1.439076000

B -5.493270000 -1.593919000 0.000126000

B -3.975988000 -1.319726000 0.880634000

B -3.050646000 -0.089213000 0.000115000

B -3.951773000 0.397851000 -1.429638000

B -5.475060000 -0.527261000 -1.439037000

C -6.072252000 -0.951236000 2.778083000

C -3.167034000 0.568734000 2.739262000

C -6.080195000 2.293673000 1.728430000

C -3.094883000 2.744065000 -0.000130000

C -6.079958000 2.293390000 -1.728968000

C -3.166812000 0.568322000 -2.739216000

C -6.071959000 -0.951711000 -2.778039000

C -3.222185000 -2.248096000 -1.842989000

C -6.158129000 -2.966523000 0.000234000

C -3.222221000 -2.247771000 1.843425000

C -7.929660000 -0.023005000 -0.000164000

N -2.521336000 0.382220000 -3.672878000

N -2.384906000 3.649988000 -0.000187000

N -6.528919000 3.129750000 2.379130000

N -2.521590000 0.382613000 3.672943000

N -2.603516000 -2.669175000 2.716498000

N -6.615332000 -4.022419000 0.000324000

N -9.080143000 -0.016521000 -0.000238000

N -6.528567000 3.129366000 -2.379878000

N -6.436475000 -1.286149000 3.816905000

N -6.436046000 -1.286794000 -3.816854000

N -2.603856000 -2.669473000 -2.716338000

Xe 3.002136000 5.107070000 -0.000086000

B 2.228573000 0.592755000 0.902062000

B 3.103149000 -2.197925000 -0.902550000

B 1.075626000 -0.404039000 0.000140000

B 2.228489000 0.592711000 -0.901959000

B 3.763758000 0.515414000 -0.000015000

B 3.512914000 -0.520277000 1.459487000

B 1.836044000 -1.081363000 1.444233000

B 1.573854000 -2.114827000 0.000137000

B 3.103226000 -2.197899000 0.902707000

B 4.215897000 -1.186405000 0.000008000

B 3.512773000 -0.520339000 -1.459440000

B 1.835896000 -1.081408000 -1.444009000

C 0.960657000 -1.296788000 2.683844000

C 4.339614000 -0.317139000 2.722623000

C 1.747823000 1.766352000 1.744068000

C 4.853633000 1.579380000 -0.000103000

C 1.747625000 1.766278000 -1.743945000

C 4.339347000 -0.317277000 -2.722672000

C 0.960401000 -1.296950000 -2.683520000

C 3.574795000 -3.412589000 -1.691355000

C 0.473064000 -3.171353000 0.000205000

C 3.574954000 -3.412555000 1.691474000

C -0.437591000 -0.160416000 0.000159000

N 5.016190000 -0.203277000 -3.646256000

N 5.756872000 2.292178000 -0.000174000

N 1.323173000 2.597459000 2.416933000

N 5.016557000 -0.203077000 3.646127000

N 3.989697000 -4.314408000 2.273587000

N -0.451213000 -3.856974000 0.000230000

N -1.579758000 -0.109629000 0.000146000

N 1.322870000 2.597366000 -2.416767000

N 0.187486000 -1.423967000 3.521009000

N 0.187107000 -1.424273000 -3.520547000

N 3.989469000 -4.314440000 -2.273520000

Xe 6.412941000 -1.712472000 -0.000113000

Li -1.707136000 -1.450577000 -4.125460000

Li -1.706888000 -1.449757000 4.125504000

**Supplementary Table 21.** Cartesian Coordinates (in Å) of TS Li_2_[B_12_CN_11_···Xe]···[B_12_CN_11_-Xe] on B3LYP-GD3BJ/6-311++G(2d,2p) (SDD) level of theory.

H_0K_ = ‑2686.708906 Ha

Atom X Y Z

B 5.737957000 0.051498000 1.225811000

B 3.480849000 0.830485000 -1.199827000

B 6.037935000 1.351083000 0.030959000

B 6.024101000 -0.355369000 -0.496432000

B 4.770552000 -1.228077000 0.436264000

B 3.972200000 -0.037759000 1.513202000

B 4.781549000 1.557383000 1.271258000

B 4.473953000 2.089570000 -0.404568000

B 3.204009000 1.250822000 0.506675000

B 3.257868000 -0.431608000 -0.018703000

B 4.434095000 -0.700626000 -1.267297000

B 5.233829000 0.895471000 -1.499623000

C 4.835494000 2.524639000 2.450348000

C 3.200621000 -0.258019000 2.822758000

C 6.710684000 -0.299854000 2.345317000

C 4.983542000 -2.680632000 0.841976000

C 7.274179000 -1.087891000 -0.967504000

C 4.216606000 -1.681069000 -2.413772000

C 5.727181000 1.303356000 -2.884627000

C 2.493305000 1.195929000 -2.314649000

C 4.266945000 3.547521000 -0.803675000

C 2.013540000 1.913828000 1.207214000

C 7.320143000 2.175668000 0.050052000

N 3.984471000 -2.402992000 -3.279039000

N 5.165321000 -3.766848000 1.174652000

N 7.413085000 -0.567824000 3.216350000

N 2.439653000 -0.176178000 3.681264000

N 1.235294000 2.260762000 1.978584000

N 4.040883000 4.626915000 -1.132283000

N 8.280073000 2.809447000 0.064466000

N 8.209047000 -1.650123000 -1.333156000

N 4.788432000 3.219335000 3.366383000

N 6.029375000 1.616267000 -3.949808000

N 1.767920000 1.494333000 -3.150746000

Xe 1.812811000 -3.007472000 0.026882000

B -2.816339000 -1.270014000 0.630123000

B -4.298400000 1.479497000 -0.759229000

B -1.899819000 0.024079000 -0.172620000

B -2.923681000 -1.055767000 -1.146638000

B -4.389917000 -1.389930000 -0.193467000

B -4.245845000 -0.511134000 1.378285000

B -2.699868000 0.358147000 1.371352000

B -2.722506000 1.586010000 0.067817000

B -4.187286000 1.264479000 1.028212000

B -5.149413000 0.175563000 0.043682000

B -4.423484000 -0.161932000 -1.515020000

B -2.878032000 0.702165000 -1.479764000

C -1.804067000 0.569538000 2.598695000

C -4.943442000 -1.023981000 2.632412000

C -2.077674000 -2.454809000 1.245019000

C -5.230860000 -2.656599000 -0.295963000

C -2.275031000 -2.039425000 -2.115082000

C -5.266641000 -0.365690000 -2.768188000

C -2.133812000 1.228602000 -2.715894000

C -5.033357000 2.666987000 -1.369373000

C -1.914857000 2.877783000 0.131278000

C -4.836558000 2.263014000 1.978832000

C -0.372788000 -0.041670000 -0.269271000

N -5.950471000 -0.495492000 -3.684565000

N -5.936865000 -3.562734000 -0.364290000

N -1.455196000 -3.310098000 1.697373000

N -5.529204000 -1.383464000 3.555373000

N -5.386536000 2.989101000 2.682071000

N -1.261994000 3.825242000 0.126372000

N 0.771308000 -0.103163000 -0.322989000

N -1.719574000 -2.736228000 -2.843138000

N -1.009193000 0.723984000 3.411186000

N -1.453727000 1.593719000 -3.564444000

N -5.639787000 3.532737000 -1.824276000

Xe -7.403161000 0.273855000 0.197278000

Li 0.306465000 1.945141000 -4.302579000

Li 0.897611000 1.196503000 3.732674000

**Supplementary Table 22.** Cartesian Coordinates (in Å) of [F_5_C_6_Xe]^+^AsF_6_^-^ on B3LYP-GD3BJ/6-311++G(2d,2p) (SDD) level of theory.

H_0K_ = ‑3578.644909 Ha

Atom X Y Z

C 2.079817000 0.027640000 -0.183430000

C 2.702224000 -1.206665000 -0.086425000

C 4.064544000 -1.263391000 0.174979000

C 4.784509000 -0.084537000 0.336002000

C 4.152735000 1.150441000 0.237204000

C 2.790579000 1.206437000 -0.024339000

F 2.013528000 -2.331163000 -0.241617000

F 4.678459000 -2.434698000 0.269814000

F 6.082817000 -0.138298000 0.584178000

F 4.850149000 2.267622000 0.391207000

F 2.185698000 2.385180000 -0.119761000

Xe -0.001433000 0.112646000 -0.560225000

As -3.523455000 -0.038429000 0.256187000

F -4.427937000 1.344302000 -0.257168000

F -2.416800000 1.013458000 1.148689000

F -4.500538000 -0.322350000 1.646692000

F -2.433787000 -1.374353000 0.648917000

F -4.445489000 -1.086439000 -0.766055000

F -2.374441000 0.255149000 -1.185559000

**Supplementary Table 23.** Cartesian Coordinates (in Å) of TS [F_5_C_6_Xe]^+^AsF_6_^-^ on B3LYP-GD3BJ/6-311++G(2d,2p) (SDD) level of theory.

H_0K_ = ‑3578.617035 Ha

Atom X Y Z

C 1.030851000 0.423502000 -0.059977000

C 1.266078000 -0.162236000 1.157669000

C 2.396733000 -0.978093000 1.227680000

C 3.200840000 -1.178512000 0.112895000

C 2.899814000 -0.560764000 -1.094796000

C 1.772531000 0.249325000 -1.203961000

F 0.563822000 0.097252000 2.241444000

F 2.742650000 -1.496191000 2.397432000

F 4.280297000 -1.942882000 0.209834000

F 3.648836000 -0.783290000 -2.167142000

F 1.425240000 0.778416000 -2.362462000

Xe -0.202476000 2.568792000 0.201263000

As -2.103307000 -1.141266000 -0.149857000

F -1.935352000 -2.129452000 -1.562224000

F -3.253248000 -0.126157000 -0.963567000

F -3.295857000 -2.141208000 0.580505000

F -2.077691000 -0.007745000 1.189787000

F -0.768100000 -1.994797000 0.598907000

F -0.781518000 -0.010869000 -0.913623000

**Supplementary Table 24.** Cartesian Coordinates (in Å) of the starting geometry in Supplementary Figure 2 on B3LYP-GD3BJ/6-311++G(2d,2p) (SDD) level of theory.

H_0K_ = ‑3498.711274 Ha

Atom X Y Z

B 2.771585000 1.416963000 1.452477000

B 3.784470000 -0.045308000 -1.459647000

B 3.195982000 2.372692000 -0.000345000

B 4.473954000 1.506050000 0.897829000

B 3.788260000 -0.044741000 1.458429000

B 2.083840000 -0.129784000 0.899076000

B 1.730387000 1.361408000 0.001827000

B 2.767763000 1.416380000 -1.451612000

B 2.081589000 -0.130161000 -0.895766000

B 3.359005000 -0.906399000 0.000137000

B 4.843514000 0.010897000 -0.002020000

B 4.471593000 1.505696000 -0.901532000

C 0.334044000 1.985060000 0.003660000

C 1.016484000 -0.935533000 1.642007000

C 2.257197000 2.047799000 2.745540000

C 4.212515000 -0.816778000 2.705896000

C 5.545874000 2.228032000 1.709413000

C 6.186594000 -0.715310000 -0.003660000

C 5.541352000 2.227354000 -1.716251000

C 4.205319000 -0.817852000 -2.707955000

C 2.249591000 2.046522000 -2.743517000

C 1.012873000 -0.936357000 -1.636197000

C 3.082896000 3.895748000 -0.000562000

N 7.142065000 -1.356813000 -0.004839000

N 4.496979000 -1.488498000 3.596104000

N 1.813692000 2.489634000 3.711009000

N 0.208973000 -1.587687000 2.139024000

N 0.204880000 -1.588780000 -2.132053000

N 1.802955000 2.487707000 -3.707841000

N 2.967793000 5.040428000 -0.000752000

N 6.354253000 2.759081000 2.332511000

N -0.707922000 2.470604000 0.005293000

N 6.348020000 2.758146000 -2.341782000

N 4.487302000 -1.489884000 -3.598717000

Xe 3.521891000 -3.176661000 0.000370000

C -3.081365000 0.025958000 1.158797000

C -3.425705000 1.340672000 0.716399000

C -3.424643000 1.342000000 -0.711612000

C -3.079856000 0.028049000 -1.156040000

C -2.868759000 -0.788329000 0.000802000

Co -4.786500000 -0.030675000 0.000222000

C -3.665366000 2.524102000 1.588057000

C -3.662574000 2.526982000 -1.581593000

C -2.465240000 -2.223542000 0.000457000

C -2.926712000 -0.400703000 2.578838000

C -6.702959000 0.729805000 -0.021101000

C -6.486498000 -0.101982000 -1.164991000

C -6.148907000 -1.412948000 -0.701218000

C -6.156088000 -1.391187000 0.729361000

C -6.498113000 -0.066767000 1.149684000

C -6.628892000 0.315060000 -2.591416000

C -5.877765000 -2.603590000 -1.560343000

C -6.655105000 0.393569000 2.561197000

C -7.114597000 2.164710000 -0.044879000

C -5.893020000 -2.555123000 1.626722000

C -2.924028000 -0.395832000 -2.576768000

H -6.381825000 1.365253000 -2.728939000

H -7.658684000 0.173618000 -2.926269000

H -5.988985000 -0.271088000 -3.246648000

H -5.207739000 -3.306989000 -1.071778000

H -5.433386000 -2.319513000 -2.511426000

H -6.807817000 -3.133044000 -1.777791000

H -5.463806000 -2.241714000 2.575457000

H -5.212901000 -3.269344000 1.168561000

H -6.823944000 -3.082400000 1.845702000

H -6.020378000 -0.170727000 3.240277000

H -7.687745000 0.259869000 2.890414000

H -6.411512000 1.447994000 2.668898000

H -6.751027000 2.701466000 0.828320000

H -8.203691000 2.245424000 -0.049075000

H -6.746090000 2.673751000 -0.932383000

H -3.098182000 -1.461626000 -2.701455000

H -1.903966000 -0.202004000 -2.908769000

H -3.598410000 0.148723000 -3.233742000

H -2.836501000 -2.744127000 0.879993000

H -1.379150000 -2.298038000 0.012602000

H -2.816758000 -2.738359000 -0.890285000

H -3.092356000 -1.468190000 2.699822000

H -3.607732000 0.136412000 3.235301000

H -1.909414000 -0.199066000 2.914792000

H -4.359917000 3.227969000 -1.128278000

H -4.041984000 2.243258000 -2.560466000

H -2.717290000 3.052639000 -1.724539000

H -4.036211000 2.237769000 2.569349000

H -4.370567000 3.220576000 1.139672000

H -2.722541000 3.055898000 1.724367000

**Supplementary Table 25.** Cartesian Coordinates (in Å) of the TS in Supplementary Figure 2 on B3LYP-GD3BJ/6-311++G(2d,2p) (SDD) level of theory.

H_0K_ = ‑3498.661539 Ha

Atom X Y Z

B -4.214933000 -1.411040000 1.261448000

B -2.910050000 0.505848000 -1.283788000

B -5.036546000 -1.206799000 -0.315038000

B -4.925361000 0.176953000 0.809934000

B -3.322849000 0.106665000 1.619180000

B -2.450578000 -1.358393000 0.965483000

B -3.526319000 -2.151100000 -0.227574000

B -3.808430000 -1.017784000 -1.598744000

B -2.202030000 -1.110183000 -0.822660000

B -2.286568000 0.195191000 0.271553000

B -3.620122000 1.258056000 0.225102000

B -4.673054000 0.421432000 -0.960543000

C -3.436392000 -3.659956000 -0.429007000

C -1.397141000 -2.096847000 1.785508000

C -4.767396000 -2.262956000 2.398096000

C -2.965395000 0.640531000 3.000886000

C -6.127548000 0.761313000 1.541486000

C -3.613099000 2.764497000 0.442928000

C -5.646369000 1.222333000 -1.817057000

C -2.188513000 1.343419000 -2.334228000

C -3.980522000 -1.516616000 -3.028999000

C -0.943470000 -1.635277000 -1.506402000

C -6.370714000 -1.889413000 -0.600806000

N -3.595174000 3.902597000 0.608474000

N -2.616552000 1.050883000 4.017677000

N -5.156681000 -2.906084000 3.269252000

N -0.561233000 -2.629501000 2.369055000

N 0.029794000 -2.012674000 -1.988446000

N -4.069310000 -1.888500000 -4.114320000

N -7.373427000 -2.409266000 -0.818732000

N -7.022598000 1.214019000 2.105365000

N -3.317430000 -4.794574000 -0.579268000

N -6.361132000 1.841975000 -2.472180000

N -1.558525000 1.953666000 -3.078886000

Xe -0.020913000 3.550566000 0.109449000

C 3.754101000 0.200713000 1.903671000

C 4.896624000 -0.656625000 1.970805000

C 4.524009000 -1.929985000 1.434026000

C 3.151460000 -1.863545000 1.037494000

C 2.680398000 -0.544064000 1.322639000

Co 4.273137000 -0.446233000 0.015690000

C 6.220887000 -0.311103000 2.565365000

C 5.395183000 -3.139409000 1.367389000

C 1.295164000 -0.037113000 1.111983000

C 3.669657000 1.600237000 2.414155000

C 5.769140000 0.506965000 -1.032349000

C 5.577182000 -0.818738000 -1.535577000

C 4.226385000 -0.927506000 -1.997182000

C 3.585174000 0.333275000 -1.782813000

C 4.535973000 1.216642000 -1.181869000

C 6.621818000 -1.880304000 -1.628419000

C 3.614177000 -2.122414000 -2.648362000

C 4.295897000 2.649587000 -0.841790000

C 7.047362000 1.072808000 -0.509680000

C 2.196136000 0.693334000 -2.187571000

C 2.337942000 -2.985447000 0.489454000

H 7.369149000 -1.782592000 -0.844030000

H 7.141557000 -1.809766000 -2.586237000

H 6.191133000 -2.876440000 -1.564820000

H 2.539297000 -2.164311000 -2.497751000

H 4.048214000 -3.048793000 -2.278225000

H 3.793166000 -2.086435000 -3.725324000

H 1.752903000 1.424911000 -1.517390000

H 1.547779000 -0.175461000 -2.228921000

H 2.205755000 1.139539000 -3.184268000

H 3.256837000 2.830972000 -0.578158000

H 4.526827000 3.280749000 -1.702504000

H 4.919779000 2.981725000 -0.015445000

H 6.870972000 1.859700000 0.220268000

H 7.624816000 1.509521000 -1.327205000

H 7.667404000 0.311148000 -0.042970000

H 1.620617000 -2.646425000 -0.251753000

H 1.769709000 -3.450341000 1.296720000

H 2.961839000 -3.748726000 0.031394000

H 1.293213000 1.027015000 0.899790000

H 0.705117000 -0.204425000 2.013647000

H 0.814175000 -0.558282000 0.288924000

H 2.952504000 2.193275000 1.852043000

H 4.632358000 2.105006000 2.375157000

H 3.342870000 1.596442000 3.456021000

H 6.444721000 -2.877879000 1.255147000

H 5.116815000 -3.790524000 0.542355000

H 5.296782000 -3.719384000 2.287281000

H 6.440028000 0.750244000 2.477570000

H 7.031204000 -0.864524000 2.095894000

H 6.228445000 -0.560242000 3.628526000

**Supplementary Table 26.** Cartesian Coordinates (in Å) of the reaction product in Supplementary Figure 2 on B3LYP-GD3BJ/6-311++G(2d,2p) (SDD) level of theory.

H_0K_ = ‑3498.750919 Ha

Atom X Y Z

B -3.520436000 -0.271505000 -1.607546000

B -2.328002000 -0.839527000 1.445541000

B -4.611640000 -0.667791000 -0.246600000

B -3.496858000 -1.910779000 -0.898797000

B -1.956953000 -1.107239000 -1.331321000

B -2.124946000 0.622334000 -0.958803000

B -3.756393000 0.909512000 -0.285345000

B -3.882471000 -0.003723000 1.233017000

B -2.340001000 0.788005000 0.788108000

B -1.188850000 -0.437089000 0.142711000

B -2.071837000 -2.016679000 0.175826000

B -3.720842000 -1.752019000 0.851813000

C -4.474566000 2.243559000 -0.477896000

C -1.363562000 1.694562000 -1.743377000

C -4.029963000 -0.014377000 -3.024167000

C -1.124832000 -1.685701000 -2.480085000

C -3.986786000 -3.144616000 -1.654045000

C -1.343633000 -3.344388000 0.400446000

C -4.339035000 -2.803351000 1.774848000

C -1.965749000 -1.087978000 2.908059000

C -4.632831000 0.442792000 2.488893000

C -1.717825000 1.908851000 1.628414000

C -6.126311000 -0.776659000 -0.403976000

N -0.768302000 -4.322832000 0.589839000

N -0.472383000 -2.163379000 -3.298539000

N -4.385934000 0.184341000 -4.100163000

N -0.709793000 2.471426000 -2.285109000

N -1.138725000 2.639471000 2.304232000

N -5.091258000 0.732556000 3.504218000

N -7.269362000 -0.861355000 -0.504374000

N -4.333726000 -4.089586000 -2.211281000

N -4.989155000 3.264875000 -0.604932000

N -4.707570000 -3.565309000 2.554951000

N -1.789584000 -1.269900000 4.023053000

C 0.360530000 -0.122697000 0.414883000

C 1.372127000 -1.066221000 -0.139415000

H 0.544271000 0.873456000 0.014813000

H 0.506621000 -0.028992000 1.490713000

C 1.930732000 -2.190570000 0.549169000

C 1.978288000 -0.993492000 -1.436141000

C 2.842208000 -2.842411000 -0.339219000

C 1.634581000 -2.621953000 1.947555000

C 2.868331000 -2.106277000 -1.562579000

C 1.760880000 0.048622000 -2.480652000

C 3.583111000 -4.103726000 -0.050575000

H 2.538622000 -2.939024000 2.463121000

H 0.943377000 -3.462443000 1.941523000

H 1.187021000 -1.813936000 2.522784000

C 3.636200000 -2.460823000 -2.789623000

H 2.692969000 0.296636000 -2.982778000

H 1.337657000 0.959886000 -2.071251000

H 1.072572000 -0.319280000 -3.237909000

H 4.513380000 -4.165153000 -0.610875000

H 2.968057000 -4.960516000 -0.332835000

H 3.810494000 -4.203703000 1.008478000

H 3.924941000 -1.576227000 -3.352600000

H 3.006712000 -3.067284000 -3.443929000

H 4.530971000 -3.034844000 -2.559841000

Co 3.427623000 -0.895115000 0.015448000

C 5.472440000 -0.676723000 0.126338000

C 4.894375000 0.465516000 -0.512879000

C 4.916172000 -0.776967000 1.440695000

C 6.510805000 -1.575777000 -0.459028000

C 3.971126000 1.064724000 0.399877000

C 5.241432000 0.975102000 -1.871855000

C 3.985237000 0.296383000 1.607920000

C 5.288435000 -1.791169000 2.470914000

H 6.426933000 -1.632207000 -1.541890000

H 6.439325000 -2.585319000 -0.060216000

H 7.510292000 -1.201813000 -0.226953000

C 3.163977000 2.295778000 0.155093000

H 4.439106000 1.574203000 -2.294000000

H 5.464496000 0.165403000 -2.563584000

H 6.128178000 1.610068000 -1.816344000

C 3.200102000 0.599052000 2.841384000

H 5.546227000 -2.747605000 2.021224000

H 4.484088000 -1.956470000 3.183717000

H 6.158597000 -1.451223000 3.036460000

H 2.873885000 2.393810000 -0.887884000

H 3.741491000 3.184282000 0.417642000

H 2.258674000 2.314802000 0.755670000

H 2.262090000 1.098456000 2.609492000

H 3.767566000 1.265667000 3.494019000

H 2.979142000 -0.302545000 3.408254000

H -1.780766000 -1.422365000 5.014528000

Xe 0.531998000 5.157377000 -0.009940000

**Supplementary Table 27.** Cartesian Coordinates (in Å) of (CoCp^*^_2_)_2_[B_12_(CN)_11_Xe]_2_ on B3LYP-GD3BJ/6-311G (SDD) level of theory.

H_0K_ = ‑6995.982357 Ha

Atom X Y Z

B -1.678832000 -5.882126000 -1.603737000

B 0.261824000 -4.512500000 0.862059000

B -1.343073000 -6.744298000 -0.060861000

B -0.022810000 -6.497136000 -1.254258000

B -0.249459000 -4.879128000 -1.993093000

B -1.709373000 -4.122880000 -1.249756000

B -2.375566000 -5.278743000 -0.060246000

B -1.165231000 -5.520263000 1.244132000

B -1.389824000 -3.897136000 0.513175000

B -0.104033000 -3.725965000 -0.670126000

B 0.964420000 -5.118944000 -0.682852000

B 0.292401000 -6.271202000 0.506465000

C -3.871320000 -5.293771000 0.216516000

C -2.578919000 -3.116812000 -1.987233000

C -2.557886000 -6.464610000 -2.697134000

C 0.205134000 -4.495600000 -3.391443000

C 0.609254000 -7.633019000 -2.039965000

C 2.457017000 -4.931556000 -0.923379000

C 1.218212000 -7.189208000 1.287099000

C 1.157676000 -3.874650000 1.912010000

C -1.583553000 -5.775881000 2.682273000

C -1.994970000 -2.738676000 1.288168000

C -1.921287000 -8.120229000 0.222704000

N 3.590186000 -4.694986000 -1.058243000

N 0.588819000 -4.132648000 -4.431186000

N -3.243999000 -6.892352000 -3.536877000

N -3.251243000 -2.320651000 -2.508813000

N -2.479214000 -1.868584000 1.892171000

N -1.913760000 -5.947305000 3.787050000

N -2.370408000 -9.172977000 0.443143000

N 1.111685000 -8.493264000 -2.645275000

N -5.016031000 -5.247520000 0.426019000

N 1.957109000 -7.863632000 1.885590000

N 1.875715000 -3.384391000 2.688356000

Xe 0.781504000 -1.731001000 -1.192464000

B -1.379907000 4.512786000 -0.626335000

B 1.983075000 4.519943000 0.012855000

B -0.463141000 3.019551000 -0.289837000

B -0.189281000 3.900552000 -1.830267000

B -0.279432000 5.648359000 -1.467625000

B -0.614835000 5.834473000 0.306471000

B -0.731076000 4.199238000 1.028792000

B 0.865683000 3.396338000 0.847449000

B 0.784999000 5.138504000 1.222741000

B 1.022180000 5.946554000 -0.322588000

B 1.328698000 4.835518000 -1.649528000

B 1.195583000 3.220517000 -0.913117000

C -1.675698000 3.906626000 2.182167000

C -1.345977000 7.058714000 0.826141000

C -2.877583000 4.461024000 -0.885874000

C -0.720566000 6.717864000 -2.449520000

C -0.623678000 3.305625000 -3.156932000

C 2.279128000 5.186029000 -2.779729000

C 1.942244000 1.984188000 -1.394945000

C 3.481233000 4.545753000 0.268453000

C 1.399430000 2.361590000 1.823983000

C 1.266840000 5.764670000 2.518576000

C -1.064380000 1.625310000 -0.327762000

N 3.035608000 5.492127000 -3.612614000

N -1.017456000 7.578205000 -3.178326000

N -4.026154000 4.364959000 -1.055074000

N -1.863225000 8.029687000 1.212302000

N 1.669758000 6.286797000 3.480164000

N 1.842124000 1.547984000 2.531303000

N -1.437527000 0.525574000 -0.410136000

N -0.961301000 2.817191000 -4.160168000

N -2.437007000 3.673400000 3.033396000

N 2.444603000 0.975553000 -1.690910000

N 4.634088000 4.542904000 0.442843000

Xe 2.021085000 7.977602000 -0.346356000

C -5.161989000 -0.019991000 -0.469864000

C -6.093674000 -1.098728000 -0.667844000

C -6.413909000 -1.656911000 0.619468000

C -5.673629000 -0.925147000 1.613189000

C -4.907815000 0.092597000 0.943219000

Co -6.917723000 0.355209000 0.561799000

C -6.564845000 -1.604065000 -1.989099000

C -7.296688000 -2.832127000 0.869416000

C -3.984723000 1.051850000 1.609295000

C -4.528345000 0.773979000 -1.559274000

C -8.767134000 0.889533000 -0.199489000

C -8.876468000 0.558210000 1.197283000

C -8.019736000 1.451736000 1.932840000

C -7.385605000 2.338109000 0.993163000

C -7.844056000 1.988474000 -0.325934000

C -9.780531000 -0.473168000 1.788283000

C -7.875145000 1.508190000 3.417942000

C -7.484330000 2.696346000 -1.591171000

C -9.534511000 0.256788000 -1.313632000

C -6.482711000 3.476472000 1.336559000

C -5.622825000 -1.228565000 3.071307000

H -9.959696000 -1.298222000 1.100805000

H -10.750728000 -0.032156000 2.033433000

H -9.370347000 -0.892412000 2.706347000

H -6.891999000 1.872628000 3.712665000

H -8.019381000 0.531828000 3.878128000

H -8.617343000 2.187706000 3.846033000

H -5.819197000 3.733366000 0.514386000

H -5.864456000 3.263726000 2.207446000

H -7.076610000 4.365485000 1.568053000

H -6.502477000 3.162265000 -1.533352000

H -8.211044000 3.486728000 -1.799484000

H -7.484127000 2.021527000 -2.446887000

H -8.995127000 0.312277000 -2.257741000

H -10.492708000 0.765031000 -1.452595000

H -9.745783000 -0.793409000 -1.115272000

H -5.431382000 -0.336172000 3.665150000

H -4.802863000 -1.923646000 3.262200000

H -6.544581000 -1.687138000 3.427537000

H -3.810757000 1.932672000 0.997048000

H -3.030343000 0.560982000 1.792676000

H -4.365281000 1.389449000 2.572284000

H -4.231571000 1.763919000 -1.223554000

H -5.196301000 0.894206000 -2.410832000

H -3.640821000 0.250068000 -1.914749000

H -8.114678000 -2.882967000 0.150625000

H -7.728601000 -2.807223000 1.868895000

H -6.720250000 -3.755308000 0.778610000

H -6.742454000 -0.792789000 -2.695169000

H -7.480346000 -2.185729000 -1.899400000

H -5.795565000 -2.243345000 -2.425568000

C 6.975388000 -1.765039000 2.553721000

C 5.714860000 -2.211025000 2.022626000

C 4.945566000 -1.044585000 1.680855000

C 5.718746000 0.121076000 2.015774000

C 6.977430000 -0.323962000 2.552161000

Co 6.727232000 -1.040404000 0.627367000

C 5.245132000 -3.622658000 1.918073000

C 3.561344000 -1.047267000 1.136740000

C 8.055778000 0.551488000 3.098997000

C 8.054050000 -2.641102000 3.098913000

C 6.277719000 -1.313859000 -1.390389000

C 6.790286000 0.011892000 -1.162475000

C 8.109653000 -0.111853000 -0.599364000

C 8.410591000 -1.514481000 -0.475384000

C 7.275867000 -2.258312000 -0.960797000

C 6.109965000 1.288709000 -1.532072000

C 9.030368000 1.020635000 -0.284387000

C 7.178616000 -3.744089000 -1.076121000

C 4.979528000 -1.649235000 -2.044599000

C 9.703636000 -2.098080000 -0.009687000

C 5.252015000 1.531589000 1.896052000

H 5.027874000 1.178462000 -1.543424000

H 6.414227000 1.597228000 -2.536204000

H 6.356604000 2.103023000 -0.852102000

H 9.738460000 0.763773000 0.502080000

H 8.483959000 1.907952000 0.033463000

H 9.609383000 1.294058000 -1.170716000

H 9.566082000 -3.083237000 0.432966000

H 10.194047000 -1.464652000 0.729057000

H 10.391559000 -2.211660000 -0.851927000

H 7.705010000 -4.249488000 -0.266598000

H 7.629205000 -4.076284000 -2.015657000

H 6.146766000 -4.088741000 -1.071213000

H 4.578261000 -2.596157000 -1.690579000

H 5.125739000 -1.745504000 -3.124458000

H 4.234383000 -0.872188000 -1.888997000

H 6.074298000 2.238193000 1.799660000

H 4.671486000 1.808294000 2.778639000

H 4.600449000 1.666161000 1.036111000

H 9.043110000 0.107150000 2.975682000

H 7.901239000 0.714046000 4.169043000

H 8.065788000 1.528630000 2.619074000

H 9.033275000 -2.168867000 3.034782000

H 8.106294000 -3.593525000 2.573238000

H 7.862859000 -2.860907000 4.152602000

H 3.360526000 -1.978054000 0.612781000

H 3.397692000 -0.217072000 0.452556000

H 2.839607000 -0.960147000 1.949310000

H 6.065655000 -4.331350000 2.012312000

H 4.741508000 -3.823194000 0.974559000

H 4.524056000 -3.828895000 2.711669000

**Supplementary Table 28.** Cartesian Coordinates (in Å) of (CoCp^*^_2_)_2_[B_12_(CN)_11_Xe]_2_ on B3LYP-GD3BJ/6-311++G(2d,2p) (SDD) level of theory. No frequency analysis was successfully conducted on this geometry.

Atom X Y Z

B 2.063571000 -5.662970000 1.238784000

B -0.211181000 -4.420067000 -0.969801000

B 1.618684000 -6.534934000 -0.257321000

B 0.429812000 -6.380046000 1.069681000

B 0.629931000 -4.762164000 1.792260000

B 1.941600000 -3.913400000 0.906953000

B 2.543477000 -5.010304000 -0.356120000

B 1.221058000 -5.325791000 -1.513212000

B 1.417695000 -3.701433000 -0.797817000

B 0.266337000 -3.618411000 0.514506000

B -0.698419000 -5.071950000 0.628463000

B -0.088549000 -6.169008000 -0.632736000

C 4.000511000 -4.922732000 -0.814443000

C 2.809893000 -2.861684000 1.598126000

C 3.106813000 -6.180330000 2.227661000

C 0.310403000 -4.405790000 3.243292000

C -0.041237000 -7.561316000 1.915161000

C -2.170414000 -4.972482000 1.042406000

C -1.044477000 -7.142007000 -1.321385000

C -1.248734000 -3.855233000 -1.939902000

C 1.490940000 -5.549666000 -2.999784000

C 1.841896000 -2.482973000 -1.615595000

C 2.255792000 -7.874899000 -0.619277000

N -3.273663000 -4.785948000 1.310638000

N 0.022455000 -4.050885000 4.299607000

N 3.911322000 -6.530987000 2.971813000

N 3.433687000 -2.048206000 2.119263000

N 2.154415000 -1.560846000 -2.227556000

N 1.697791000 -5.689121000 -4.122998000

N 2.744700000 -8.879270000 -0.894616000

N -0.421485000 -8.433589000 2.562039000

N 5.086326000 -4.792844000 -1.171315000

N -1.805972000 -7.836021000 -1.833674000

N -2.047528000 -3.434129000 -2.652397000

Xe -0.660306000 -1.668741000 1.149115000

B 1.180911000 4.607619000 0.460266000

B -2.166117000 4.370170000 -0.115869000

B 0.357795000 3.047629000 0.245595000

B 0.051910000 4.010379000 1.717584000

B 0.029702000 5.723601000 1.236747000

B 0.327738000 5.805690000 -0.542794000

B 0.534381000 4.141149000 -1.148843000

B -1.001337000 3.262786000 -0.885693000

B -1.030644000 4.968956000 -1.379672000

B -1.295719000 5.865664000 0.102716000

B -1.514430000 4.835105000 1.500591000

B -1.295010000 3.188798000 0.878751000

C 1.490583000 3.814233000 -2.294115000

C 0.972343000 7.043821000 -1.159342000

C 2.688978000 4.685516000 0.700749000

C 0.412844000 6.893863000 2.137724000

C 0.543292000 3.537458000 3.082729000

C -2.477958000 5.212183000 2.622488000

C -1.982270000 1.947415000 1.457104000

C -3.676150000 4.317225000 -0.347030000

C -1.493269000 2.124803000 -1.779278000

C -1.577234000 5.482620000 -2.708739000

C 1.067310000 1.699222000 0.352038000

N -3.242260000 5.541787000 3.417254000

N 0.636566000 7.822252000 2.779890000

N 3.827209000 4.710033000 0.864139000

N 1.389472000 8.018236000 -1.607474000

N -2.039112000 5.927638000 -3.664289000

N -1.903680000 1.249585000 -2.402935000

N 1.537898000 0.657087000 0.469511000

N 0.918388000 3.152832000 4.100228000

N 2.250451000 3.558059000 -3.119125000

N -2.453640000 0.963670000 1.822493000

N -4.817140000 4.298428000 -0.494649000

Xe -2.420808000 7.833569000 0.002981000

C 5.217897000 0.318721000 0.744603000

C 6.133109000 -0.704379000 1.140710000

C 6.463102000 -1.478691000 -0.014336000

C 5.747909000 -0.934372000 -1.127725000

C 4.986677000 0.186209000 -0.661745000

Co 6.984762000 0.495256000 -0.301469000

C 6.607273000 -0.956933000 2.530591000

C 7.365581000 -2.665192000 -0.036064000

C 4.114010000 1.053558000 -1.503274000

C 4.588761000 1.308132000 1.661675000

C 8.898462000 1.002963000 0.264791000

C 8.877228000 0.599324000 -1.107759000

C 7.992539000 1.476920000 -1.811782000

C 7.468410000 2.423544000 -0.876467000

C 8.025589000 2.128564000 0.408094000

C 9.672337000 -0.510897000 -1.711585000

C 7.699702000 1.441696000 -3.275299000

C 7.774285000 2.893108000 1.665760000

C 9.720211000 0.385343000 1.347770000

C 6.544800000 3.552246000 -1.194303000

C 5.753586000 -1.470690000 -2.518666000

H 9.880302000 -1.296728000 -0.989600000

H 10.631260000 -0.135349000 -2.075384000

H 9.155930000 -0.960146000 -2.557221000

H 6.699075000 1.808646000 -3.492547000

H 7.785352000 0.435957000 -3.679415000

H 8.404803000 2.076055000 -3.816881000

H 5.928071000 3.829084000 -0.343596000

H 5.884859000 3.313463000 -2.024365000

H 7.122237000 4.434579000 -1.479951000

H 6.803016000 3.380968000 1.651217000

H 8.531162000 3.670460000 1.791465000

H 7.818872000 2.250329000 2.542723000

H 9.255077000 0.506776000 2.322976000

H 10.704824000 0.855977000 1.391879000

H 9.874136000 -0.678285000 1.178130000

H 5.509336000 -0.701525000 -3.246595000

H 5.003612000 -2.256705000 -2.607963000

H 6.714130000 -1.910985000 -2.778520000

H 3.956208000 2.024481000 -1.043952000

H 3.146913000 0.577693000 -1.646117000

H 4.538068000 1.211922000 -2.492492000

H 4.328630000 2.229996000 1.151552000

H 5.240732000 1.554038000 2.496284000

H 3.675589000 0.878098000 2.073091000

H 8.178029000 -2.561801000 0.680813000

H 7.790347000 -2.829292000 -1.022766000

H 6.803346000 -3.563753000 0.216223000

H 6.711959000 -0.032453000 3.095106000

H 7.560416000 -1.479611000 2.544185000

H 5.873452000 -1.575671000 3.048564000

C -6.993952000 -2.117234000 -2.249175000

C -5.730719000 -2.520309000 -1.712363000

C -4.986985000 -1.335815000 -1.414182000

C -5.780065000 -0.203911000 -1.778952000

C -7.024283000 -0.686548000 -2.291604000

Co -6.751301000 -1.336439000 -0.354664000

C -5.253983000 -3.922184000 -1.541946000

C -3.616319000 -1.281148000 -0.841424000

C -8.131530000 0.152311000 -2.836486000

C -8.065721000 -3.031164000 -2.741631000

C -6.271970000 -1.519533000 1.655457000

C -6.832563000 -0.230404000 1.390279000

C -8.145266000 -0.421440000 0.852780000

C -8.394169000 -1.829103000 0.783539000

C -7.233916000 -2.508204000 1.276141000

C -6.184119000 1.081896000 1.683509000

C -9.102950000 0.663678000 0.487400000

C -7.071129000 -3.984548000 1.424337000

C -4.945030000 -1.788589000 2.281947000

C -9.661214000 -2.476275000 0.332143000

C -5.353293000 1.220102000 -1.681964000

H -5.100338000 1.008247000 1.656782000

H -6.461426000 1.417461000 2.685202000

H -6.489041000 1.856248000 0.983722000

H -9.790841000 0.350522000 -0.294478000

H -8.584216000 1.556651000 0.145345000

H -9.700474000 0.946250000 1.356796000

H -9.479909000 -3.460296000 -0.093271000

H -10.178887000 -1.875140000 -0.412558000

H -10.339820000 -2.605449000 1.178000000

H -7.614302000 -4.529009000 0.654586000

H -7.464585000 -4.306404000 2.391098000

H -6.027186000 -4.283441000 1.381188000

H -4.517468000 -2.727372000 1.940030000

H -5.062487000 -1.863100000 3.365625000

H -4.237849000 -0.986867000 2.089796000

H -6.194112000 1.897513000 -1.557668000

H -4.820112000 1.510453000 -2.588416000

H -4.671974000 1.374506000 -0.851175000

H -9.101961000 -0.320357000 -2.699317000

H -7.990774000 0.305976000 -3.908448000

H -8.160861000 1.132844000 -2.367780000

H -9.051088000 -2.577402000 -2.665675000

H -8.079020000 -3.968736000 -2.190515000

H -7.894730000 -3.274308000 -3.792360000

H -3.396791000 -2.193587000 -0.296187000

H -3.501169000 -0.432373000 -0.172357000

H -2.885317000 -1.179438000 -1.641791000

H -6.081455000 -4.622709000 -1.461823000

H -4.628105000 -4.037187000 -0.661754000

H -4.649005000 -4.208194000 -2.403320000

**Supplementary Table 29.** Cartesian Coordinates (in Å) of [B_12_(CN)_11_-Xe···N_2_]^-^ on BMK-GD3BJ/aug-cc-pVTZ (SDD) level of theory.

H_0K_ = ‑1444.529005 Ha

Atom X Y Z

B 1.410686000 0.784813000 -1.535847000

B 0.350991000 -0.627474000 1.537885000

B 2.436954000 0.606140000 0.000000000

B 1.054098000 1.841207000 -0.000005000

B -0.227653000 1.083994000 -0.949565000

B 0.350995000 -0.627482000 -1.537885000

B 1.988738000 -0.924774000 -0.948854000

B 1.988734000 -0.924777000 0.948863000

B 0.708270000 -1.685318000 0.000004000

B -0.574195000 -0.413062000 0.000000000

B -0.227655000 1.084001000 0.949555000

B 1.410687000 0.784817000 1.535839000

C 3.044633000 -1.690905000 -1.721484000

C -0.370857000 -1.270858000 -2.706909000

C 1.993906000 1.412670000 -2.786549000

C -1.385543000 1.746126000 -1.672636000

C 1.343962000 3.329426000 -0.000004000

C -1.385546000 1.746131000 1.672624000

C 1.993897000 1.412683000 2.786541000

C -0.370858000 -1.270843000 2.706915000

C 3.044636000 -1.690897000 1.721493000

C 0.261074000 -3.134974000 0.000004000

C 3.879499000 1.093223000 0.000000000

N -2.319897000 2.173552000 2.182995000

N -2.319893000 2.173549000 -2.183004000

N 2.414586000 1.885164000 -3.743096000

N -1.002095000 -1.763726000 -3.528455000

N -0.175268000 -4.196059000 0.000005000

N 3.827383000 -2.285009000 2.312707000

N 4.966281000 1.459967000 0.000000000

N 1.539664000 4.459419000 -0.000003000

N 3.827371000 -2.285026000 -2.312699000

N 2.414566000 1.885184000 3.743090000

N -1.002091000 -1.763709000 3.528467000

Xe -2.685180000 -1.125047000 0.000003000

N -4.853860000 2.919921000 -0.000016000

N -4.551944000 1.872690000 -0.000003000

**Supplementary Table 30.** Cartesian Coordinates (in Å) of TS [B_12_(CN)_11_···Xe···N_2_]^-^ on BMK-GD3BJ/aug-cc-pVTZ (SDD) level of theory.

H_0K_ = -1444.487750 Ha

Atom X Y Z

B -1.563783000 -0.238732000 1.639026000

B -0.348018000 0.277744000 -1.621179000

B -2.586820000 -0.563619000 0.008388000

B -2.207581000 1.090244000 0.620349000

B -0.417689000 1.090613000 1.278103000

B 0.128571000 -0.742451000 1.221800000

B -1.248388000 -1.680672000 0.493599000

B -1.466781000 -1.114665000 -1.326118000

B 0.123350000 -1.306488000 -0.613284000

B 0.402585000 0.363794000 -0.072315000

B -0.869469000 1.688639000 -0.377752000

B -2.054315000 0.673579000 -1.226723000

C -1.508627000 -3.119860000 0.894657000

C 1.137163000 -1.285565000 2.207221000

C -2.169915000 -0.542220000 2.996064000

C 0.185096000 2.029401000 2.298871000

C -3.229613000 2.081582000 1.138476000

C -0.561213000 3.107994000 -0.806933000

C -3.024882000 1.239294000 -2.242982000

C 0.384737000 0.599996000 -2.906833000

C -1.986039000 -2.044409000 -2.403292000

C 1.153799000 -2.284653000 -1.130619000

C -4.023026000 -1.047422000 0.122574000

N -0.335868000 4.183495000 -1.134294000

N 0.673605000 2.742092000 3.052798000

N -2.612995000 -0.767314000 4.029572000

N 1.913145000 -1.690073000 2.948122000

N 1.906688000 -3.048439000 -1.536143000

N -2.365893000 -2.747133000 -3.226049000

N -5.107289000 -1.411121000 0.206803000

N -3.990710000 2.843441000 1.532574000

N -1.685800000 -4.211465000 1.198556000

N -3.749903000 1.673314000 -3.018221000

N 0.976355000 0.851045000 -3.856621000

Xe 3.841447000 -0.374142000 -0.037029000

N 2.886775000 3.363761000 -0.007837000

N 2.200275000 2.519668000 -0.063993000

**Supplementary Table 31.** Cartesian Coordinates (in Å) of [B_12_(CN)_11_-N_2_···Xe]^-^ on BMK-GD3BJ/aug-cc-pVTZ (SDD) level of theory.

H_0K_ = -1444.540528 Ha

Atom X Y Z

B -2.559540000 0.712451000 0.949946000

B 0.063616000 -0.737443000 -0.952453000

B -2.643307000 -0.877687000 -0.000195000

B -2.559626000 0.712997000 -0.949470000

B -1.495210000 1.746840000 0.000486000

B -0.898360000 0.797936000 1.541573000

B -1.601528000 -0.817909000 1.535196000

B -1.010395000 -1.762865000 -0.000517000

B 0.063698000 -0.738018000 0.951961000

B 0.064215000 0.805412000 0.000175000

B -0.898498000 0.798779000 -1.541176000

B -1.601665000 -0.817049000 -1.535633000

C -2.023949000 -1.564980000 2.784411000

C -0.345500000 1.591837000 2.709207000

C -3.761866000 1.215043000 1.723789000

C -1.386752000 3.259280000 0.000889000

C -3.762006000 1.216004000 -1.722957000

C -0.345639000 1.593285000 -2.708400000

C -2.024118000 -1.563489000 -2.785214000

C 1.323947000 -1.134629000 -1.695747000

C -0.952797000 -3.277417000 -0.000942000

C 1.324038000 -1.135640000 1.694997000

C -3.932725000 -1.686405000 -0.000354000

N 0.139053000 2.216508000 -3.540634000

N -1.216071000 4.393683000 0.001189000

N -4.661748000 1.610819000 2.314278000

N 0.139174000 2.214619000 3.541781000

N 2.299331000 -1.386312000 2.244280000

N -0.888331000 -4.422377000 -0.001266000

N -4.904541000 -2.295618000 -0.000472000

N -4.661941000 1.612101000 -2.313152000

N -2.327569000 -2.124135000 3.738479000

N -2.327759000 -2.122165000 -3.739556000

N 2.299265000 -1.384969000 -2.245135000

Xe 4.796439000 -0.104571000 0.000013000

N 2.193727000 2.263656000 0.000189000

N 1.303055000 1.631660000 0.000274000

**Supplementary Table 32.** Cartesian Coordinates (in Å) of CH_3_-Xe···N_2_^+^ on BMK-GD3BJ/aug-cc-pVTZ (SDD) level of theory.

H_0K_ = -164.417660 Ha

Atom X Y Z

C 0.927590000 -0.029930000 0.000303000

H 1.172939000 0.508856000 0.908609000

H 1.149723000 -1.091127000 0.015513000

H 1.172403000 0.482314000 -0.923370000

Xe -1.297735000 0.003444000 -0.000028000

N 3.813475000 -0.005423000 -0.000050000

N 4.903253000 0.018788000 -0.000098000

**Supplementary Table 33.** Cartesian Coordinates (in Å) of TS CH_3_···Xe···N_2_^+^ on BMK-GD3BJ/aug-cc-pVTZ (SDD) level of theory.

H_0K_ = -164.401514 Ha

Atom X Y Z

C 1.475588000 0.000918000 0.002511000

H 1.434086000 -0.051883000 1.081756000

H 1.434053000 -0.907419000 -0.582600000

H 1.434783000 0.962125000 -0.491169000

Xe -1.284727000 -0.000083000 -0.000256000

N 3.470957000 0.000129000 0.001189000

N 4.560299000 -0.000682000 -0.002507000

**Supplementary Table 34.** Cartesian Coordinates (in Å) of CH_3_-N_2_···Xe^+^ on BMK-GD3BJ/aug-cc-pVTZ (SDD) level of theory.

H_0K_ = -164.419867 Ha

Atom X Y Z

C 2.153108000 -0.029520000 0.000074000

H 1.829212000 0.486770000 -0.906350000

H 1.829233000 0.487856000 0.905900000

H 1.861094000 -1.082086000 0.000684000

Xe -1.414396000 0.002186000 -0.000006000

N 3.593572000 0.000060000 0.000014000

N 4.683460000 0.023728000 -0.000067000
